# High-Refractive-Index Halogenated Urethane Acrylates in Polyurethane for 2‑Stage Material Applications

**DOI:** 10.1021/acsapm.5c04746

**Published:** 2026-03-17

**Authors:** Arlo Marquez-Grap, Marianela Trujillo-Lemon, Alexander J. Osterbaan, Zachary R. Mora, Andrew N. Sias, Bruce E. Kirkpatrick, Kristi S. Anseth, Christopher N. Bowman, Robert R. McLeod

**Affiliations:** † Materials Science and Engineering Program, 1877 University of Colorado Boulder, Boulder, Colorado 80303, United States; ‡ Department of Chemical and Biological Engineering, University of Colorado Boulder, Boulder, Colorado 80303, United States; § Department of Electrical, Computer, and Energy Engineering, University of Colorado Boulder, Boulder, Colorado 80309, United States

**Keywords:** high refractive index, enhanced solubility, 2-stage polymers, photopolymers, diffraction gratings, lithography

## Abstract

High-refractive-index (HRI) monomers swollen in a rubbery host matrix to form 2-stage composite materials play a crucial role in modern optics applications and are constantly being redesigned to produce enhanced material properties. To aid in this effort, we report the synthesis of two HRI halogenated acrylate monomers and characterize their affinity for high-spatial-frequency photopatterning when swollen in a polyurethane matrix. Through refractive index measurements and high-resolution photopatterning, we show that these 2-stage films support submicrometer spatial frequencies with refractive index modulation *n*_1_ on the order of 0.01 and change in index upon polymerization Δ*n* of 0.0023. Photopolymerization kinetics from FTIR fit a three-species kinetic model, where the dependence of the polymerization rate on incident light intensity reveals that the dominant termination kinetics in these 2-stage films is unimolecular termination, as opposed to the standard bimolecular. Critically, we show that photopatterning does not induce undesirable optical or mechanical properties such as haze, discoloration, stiffening of the films, or increased dispersion, making these materials suitable for photopatterning applications. High-fidelity holographic diffraction gratings and 2D photo-patterns with micron-scale features are presented to show proof of concept.

## Graphical abstract

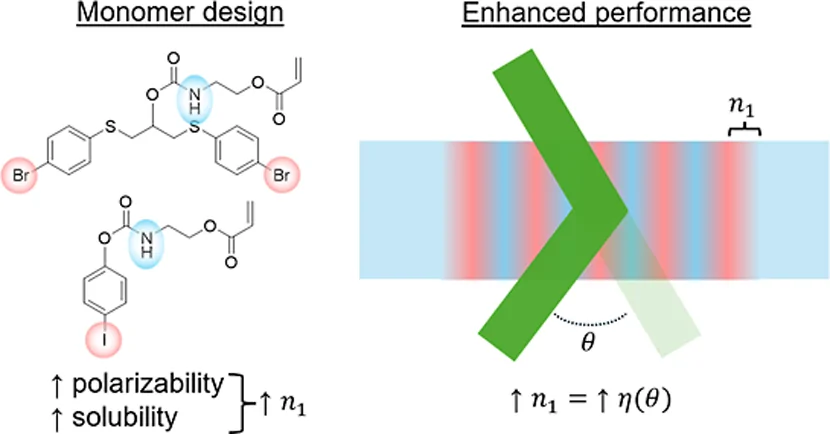

## 1 Introduction

There is general interest in the design and synthesis of high-refractive-index (HRI) monomers swollen in a rubbery host matrix to form a 2-stage material, with applications in antireflection coatings,^1,2^ optical lenses,^3–5^ light emitting diodes,^6,7^ holography,^8,9^ waveguides,^10^ nanoimprinting materials,^11,12^ and 3D printed materials.^13^ Broadly, 2-stage material systems comprise a rubbery low-index thermoset host matrix that is formed (1st stage) around an HRI monomer that is photopatterned or thermally cured (2nd stage) at a later time^14^ (Figure 1). The host matrix and HRI monomer are specifically chosen so that a significant volume fraction of the 2-stage composite consists of the 2nd-stage monomer, resulting in a large refractive index contrast between the two material stages, which is a central material design parameter in 2-stage applications. The 1st-stage host matrix typically exhibits a glass transition temperature (*T*_g_) far below room temperature,^8,15,16^ making the host matrix rubbery at room temperature, enabling relatively high transport rates during 2nd-stage polymerization, as compared to glassy host materials. This behavior is generally required for applications such as fabrication of polymer optical lenses, holographic gratings, and waveguides, where the goal is to create high-fidelity refractive index patterns through a volume by controlling the local polymerization and diffusion of the 2nd-stage HRI monomers via spatially modulated light. The change in refractive index (Δ*n*) upon polymerization of the 2nd stage is the dynamic range of a 2-stage material and is an intrinsic material design parameter created by the polymerization of the HRI monomer to a polymer.

**Figure 1:**
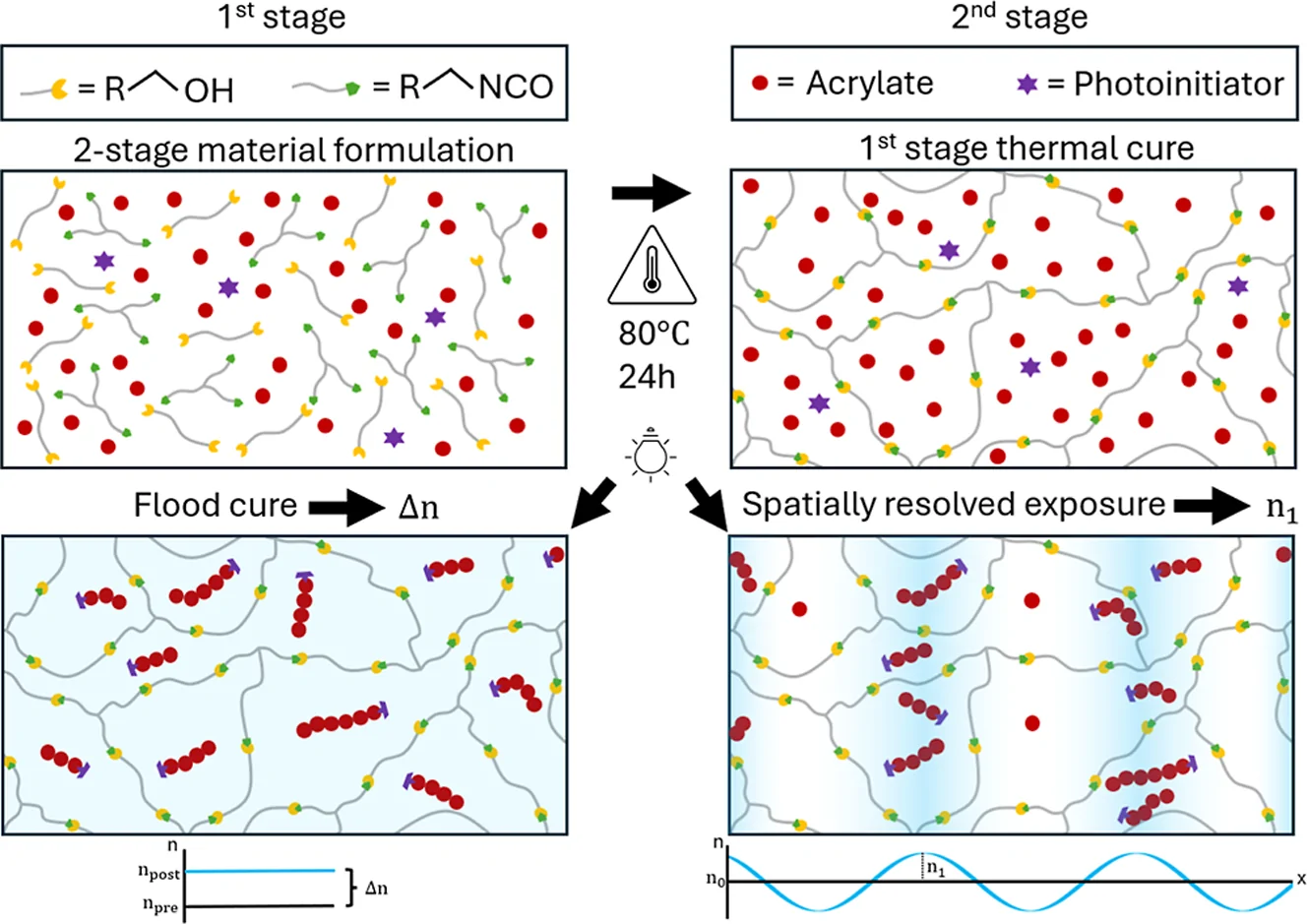
2-stage material formulation and two types of photoinduced exposure schemes that represent the intended applications. Orthogonal chemistry, e.g., 1st-stage polyurethane reaction and 2nd-stage free radical acrylate polymerization, enables a soft host matrix that defines the bulk shape and a change in refractive index upon irradiation. Full 2nd-stage polymerization with a spatially uniform light source results in a refractive index change Δ*n* that is independent of the photopatterning process and intrinsic to the 2-stage material, whereas a spatially modulated intensity pattern induces diffusion of mobile acrylate into bright regions, creating a refractive index gradient with peak to mean index modulation *n*_1_ that depends on exposure conditions and is an extrinsic 2-stage material property.

However, many 2-stage materials aim to maximize the refractive index modulation *n*_1_ over a specific length scale, which refers to the change in index upon spatially modulated polymerization. For example, after photopatterning a diffraction grating, bright regions have a higher refractive index due to a buildup of the polymer and in-diffusion of the monomer, whereas dark regions have a lower refractive index due to the out-diffusion of the HRI monomer and thus an increased concentration of the low-refractive-index host matrix. The change in refractive index from bright (exposed) to the mean is referred to as the index modulation *n*_1_ and is an extrinsic material property dependent on the photopatterning conditions, whereas the dynamic range Δ*n* is an intrinsic material property depending only on the constituent species. The weight percent ratio (wt %) of the 2nd stage to the entire formulation is often referred to as the monomer loading and is ideally maximized to facilitate a greater dynamic range and index modulation, but excessively high loading leads to deleterious effects such as phase separation or prevention of the 1st stage from forming a solid network.^8^ Therefore, 2-stage systems must be tuned to maximize the dynamic range without exceeding the maximum monomer loading, meaning that the refractive index difference between the 1st and 2nd stages, maximum loading, change in index upon 2nd-stage polymerization, and monomer mobility are key design parameters.

Transparent materials with HRI typically exhibit broad chromatic dispersion, i.e., how the index changes as a function of wavelength, which is quantified as the Abbe number.^17^ Large Abbe numbers refer to materials with low dispersion, such as 75–85 for fluorite and phosphate crown glasses, and small Abbe numbers refer to materials with high dispersion, such as 35 for many polycarbonates.^18^ For many optical systems, lenses are designed with materials that exhibit low dispersion and thus high Abbe numbers so that chromatic aberrations are minimized.^19^ Conversely, materials with low Abbe numbers are desirable if an optical element requires high dispersion, such as the dispersive prisms used in spectroscopy and for tuning lasers.^20^

The design of HRI monomers begins with understanding what affects a given species’ refractive index. According to the Lorentz–Lorenz equation (eq 1),

1 $$\frac{n^{2}-1}{n^{2}+2}=\frac{4\pi }{3}N\alpha$$

the refractive index *n* of a species is dependent upon the number density *N* in a given volume and the polarizability αof the molecule, which is a measure of the tendency of the molecule to respond to an external electromagnetic field with a dipole moment.^21^ In polymers, the polarizability α is related to the structure of the molecule and the molar refraction of the constituent elements, i.e., elements or molecules with large and diffuse electron clouds are highly polarizable because their outer electrons experience weaker nuclear attraction and are more easily distorted by external electric fields.^22^ Thus, high-index monomers can be created by combining aromatic structures, such as phenyl rings,^23^ with elemental halogens or chalcogens that have high molar refraction, such as bromine, iodine, sulfur, phosphorus, etc.^24^ HRI monomers containing sulfur are of special interest due to the dynamic properties of the thiol-X reaction.^25,26^

But, for the specific application of 2-stage materials, the inclusion of elements with high molar refraction to increase the monomer index must be balanced with the resulting decrease in the solubility of those monomers within the 1st-stage host matrix. The 2nd-stage HRI monomers are miscible in the 1st-stage host matrix if the Gibbs free energy of mixing (eq 2),

2 $$\frac{\Delta G_{mix}}{RT}=n_{1}\ln\left(\phi_{1}\right)+n_{2}\ln\left(\phi_{2}\right)+\chi_{12}n_{1}\phi_{2}$$

is negative.^27^ Because the entropy of mixing a monomer (2nd stage) into a solvent (1st stage) is always positive and thus the entropy contribution to Gibbs is always negative, the enthalpy of mixing determines the sign of Δ*G*_mix_ and is reduced (i.e., Δ*G*_mix_ *<* 0) when the Flory–Huggins interaction parameter χ_12_ is small. For a system with a highly polar solvent where hydrogen bonding is abundant, such as polyurethane, the Hansen solubility parameters determine the interaction parameter (eq 3) as

3 $$\chi_{12}=\frac{\alpha V_{1}}{RT}\left({\left(\delta_{d2}-\delta_{d1}\right)}^{2}+{0.25\left(\delta_{p2}-\delta_{p1}\right)}^{2}+{0.25\left(\delta_{h2}-\delta_{h1}\right)}^{2}\right)$$

where δ_*d*_ is the energy from dispersion forces, δ_*p*_ is the energy from dipolar intermolecular forces, δ_*h*_ is the energy from hydrogen bonds, α is a correction constant, *V*_1_ is the molar volume of the solvent, *R* is the gas constant, and *T* is the temperature.^28^ The numbers 1 and 2 in the subscripts represent the 1st-stage polyurethane solvent and 2nd-stage HRI monomer, respectively. χ_12_ is reduced when the difference between the two molecules’ energy contributions, e.g., δ_*d*2–_δ_*d*1_, is minimized, typically through similarities in the structure and elemental makeup. If a monomer swollen in a polymer network becomes immiscible, it will spontaneously phase separate into macrodomains that readily Rayleigh scatter transmitted light, producing a hazy medium.^29^ Raising the monomer index by increasing the molar refraction with halogens or chalcogens likely reduces the miscibility between the monomer and host network by increasing χ_12_, decreasing the amount of the monomer that can be present without phase separation, and ultimately reducing the desired refractive index contrast.

To counteract this drop in miscibility from the inclusion of halogen species, chemical moieties (urea and hydroxy groups) were added to the structures of the monomers to reduce the interaction parameter difference between the monomer and its surroundings, thereby increasing miscibility. In contrast, the introduction of these chemical moieties decreases the monomers’ refractive index, so an inherent trade-off between miscibility and refractive index must be balanced.

In this work, we report on the synthesis and characterization of two new urethane acrylates for applications in 2-stage material systems. Br-BPTPUA is a derivative of a previously reported^30–32^ HRI monomer and has been modified to contain pendant bromines, increasing the refractive index at the cost of reduced miscibility within polyurethane. Similarly, IPhUA was designed to contain a pendant iodine with the expectation that this pendant halogen increases the refractive index of the molecule at the cost of reduced solubility within polyurethane. Nonhalogenated BPTPUA is characterized and presented alongside the new monomers to demonstrate how pendant halogens alter chemical properties. Reduction in the solubility of Br-BPTPUA and IPhUA is justified by quantification of the Hansen solubility parameters, which predict how the structure and composition influence overall solubility.

The monomers were characterized in a 2-stage material scheme with polyurethane acting as the rubbery host matrix for two reasons. First, properties such as the refractive index cannot be measured for pure monomers or polymers of the proposed HRI species because they are powders or crystalline. More importantly, the properties measured for the neat species are different when the monomers are one component of the intended 2-stage material. For example, polymerization kinetics and refractive index are both significantly changed by the suppression of vitrification when they are swollen in a solid host matrix. In this way, the refractive index of the monomers and polymers are solvent-specific and characterization of these species in a solvent not commonly used for 2-stage applications would be irrelevant. For these reasons, HRI monomer kinetics, refractive index, absorption, and mechanics were all characterized with polyurethane acting as the rubbery host matrix. Appropriate rules of mixture, such as the Lorentz–Lorenz relation for the refractive index, are then used to extract HRI monomer properties in this environment.

## 2 Experimental Section

### 2.1 Monomer Synthesis

Commercially available reagents were used without further purification. Hydroxyethyl acrylate, 4-iodophenyl isocyanate, 4-iodophenol, 2,4,6-triiodophenol, dibutyl tin dilaurate (DBTDL), 2,2′-azobis­(2-methyl propionitrile) (AIBN), poly­(caprolactone-block-tetrahydrofuran-block-caprolactone) (average Mn 2000) (Polyol 2000), dichloromethane (DCM), toluene, hexane, and ethyl acetate (EtOAc) were purchased from Sigma-Aldrich. Epichlorohydrin was purchased from Alfa Aesar. 1,8-Diazabicyclo[5.4.0]­undec-7-ene (DBU) was purchased from Chem Impex International. Reagent-grade triethylamine (Et_3_N) was purchased from Fisher Scientific. 4-Bromobenzene thiol and isocyanate ethyl acrylate (IEA) were purchased from TCI Chemicals. The photoinitiator, diphenyl­(2,4,6-trimethylbenzoyl)­phosphine oxide (TPO), was purchased from TCI Chemicals. Covestro AG donated Desmodur N3900 polyisocyanate (TNCO), and IEA was given by Karenz.

#### 2.1.1 Synthesis of 1,3-Bis­(4-bromophenylthio)-2-propanol, Br-BPTP

To a 500 mL round-bottomed flask equipped with a magnetic stir bar were added 9 g of 4-bromothiophenol (47.6 mmol, 2.2 equiv) and 7.34 g of DBU (47.6 mmol, 2.2 equiv) in 100 mL of toluene, and the mixture was stirred for 10 min (Scheme 1A). Then, 2.00 g of epichlorohydrin (21.6 mmol, 1.0 equiv) was added dropwise. The reaction vessel was allowed to stir at room temperature for 18 h. After this period, the volatiles were removed under reduced pressure, and the residue was then diluted with dichloromethane (DCM) and washed with 1 M HCl (100 mL), distilled water (100 mL), and brine (50 mL). The combined organic extracts were dried in anhydrous Na_2_SO_4_, filtered, and concentrated under reduced pressure. The crude product was purified by flash column chromatography using 20% EtOAc in hexane as an eluent to yield 18.1 g of white solid (87%). FTIR, ^1^H nuclear magnetic resonance (NMR), ^13^C NMR, and mass spectroscopy confirmed its structure (see Supporting Information).

**Scheme 1:**
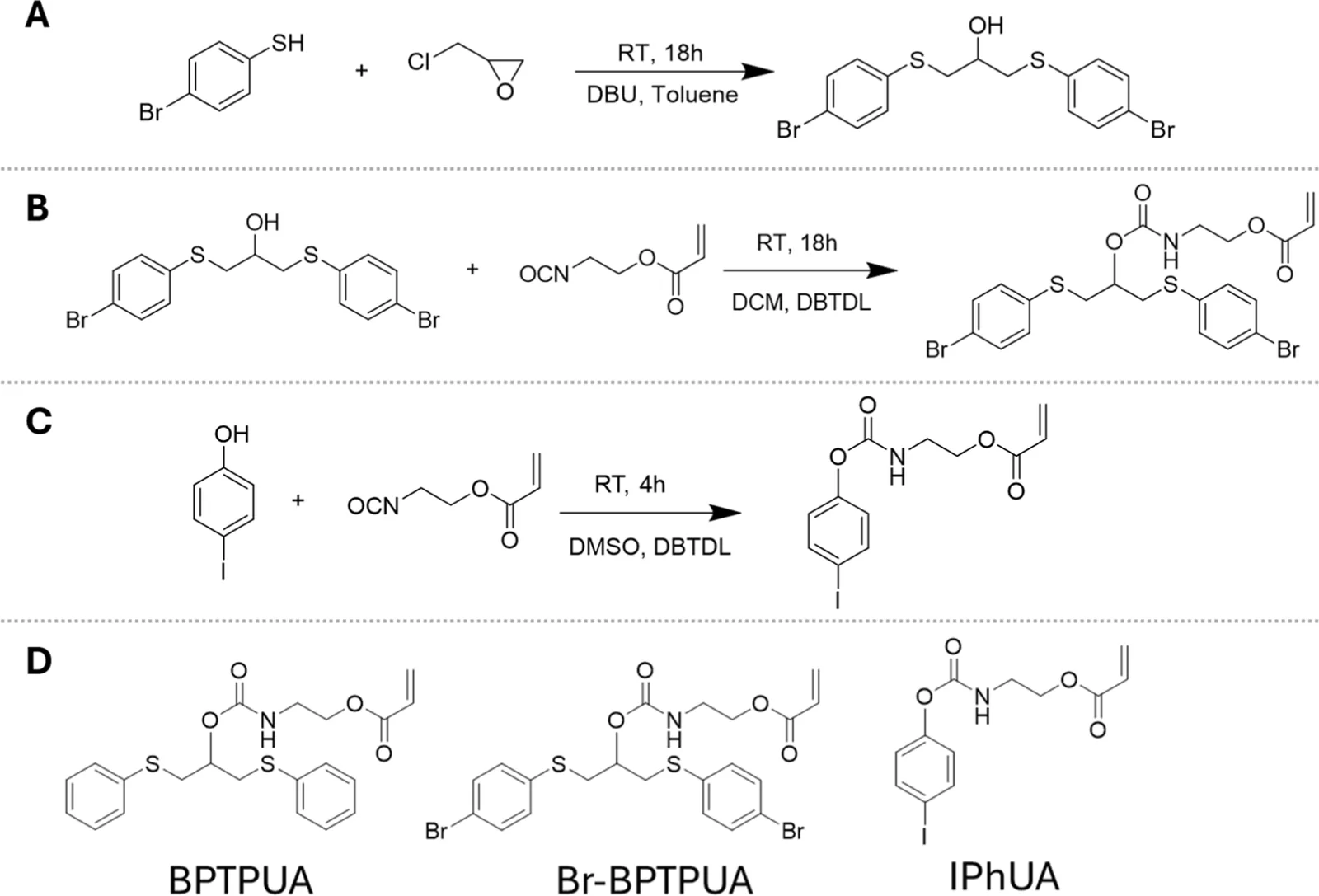
Synthesis Schemes of (A) the Precursor Br-BPTP, (B) Br-BPTPUA Monomer, and (C) IPhUA Monomer, and (D) the Chemical Structures of the HRI Acrylates^a^

a BPTPUA (synthesis reported elsewhere^32^) was characterized alongside the new monomers Br-BPTPUA and IPhUA to highlight how the presence of pendent halogens modifies material properties.

FTIR (cm^–1^): 3509 (O–H); 3080 (C–H_aromatic_); 2916 (C–H_aliphatic_); 801­(C–H).

^1^H NMR (400 MHz, CDCl_3_, 25 °C): δ 7.44–7.38 (m, 4H, C–H_aromatic_), 7.22 (d, *J* = 8.7 Hz, 4H, C–H_aromatic_), 3.86–3.75 (m, 1H, CH_2_–CH–CH_2_), 3.17 (dd, *J* = 13.8, 5.0 Hz, 2H, CH_2_), 3.05 (dd, *J* = 13.8, 7.2 Hz, 2H, CH_2_), 2.72 (br-m,1H, OH).

^13^C NMR (101 MHz, CDCl_3_, 25 °C): δ 134.2 (C_aromatic_), 132.2 (CH_aromatic_), 131.4 (CH_aromatic_), 120.7 (C_aromatic_), 68.2 (CH_2_–CH–CH_2_), 40.1 (CH_2_–CH–CH_2_).

#### 2.1.2 Synthesis of Brominated Urethane Acrylate, Br-BPTPUA

To a 100 mL round-bottomed flask equipped with a magnetic stir bar, 6.1092 g of BPTP (14 mmol, 1.0 equiv) was added, diluted with 20 mL of DCM, and stirred for 10 min under an argon atmosphere (Scheme 1B). To continue, 1.9857 g (14 mmol, 1.0 equiv) of IEA was added dropwise to the flask under a nitrogen atmosphere, followed by 2 drops of DBTDL. The reaction mixture was allowed to stir at room temperature for 24 h. Mid-IR was used to verify reaction completion following the disappearance of the isocyanate peak at ∼2250 cm^–1^. After this period, the volatiles were removed under reduced pressure. The crude product was used without further purification and stored in an EtOAc solution in the refrigerator to avoid polymerization. The title compound, Br-BPTPUA, was obtained as a white solid (100% yield). FTIR, ^1^H NMR, ^13^C NMR, and mass spectroscopy confirmed its structure (see Supporting Information).

FTIR (cm^–1^): 3351 (N–H); 3050 (C–H); 2952 (C–H); 1712 (C=O); 1634,1618 (C=C); 801 (C–H).

^1^H NMR (400 MHz, CDCl_3_, 25 °C) δ 7.37 (d, *J* = 8.7 Hz, 4H, C–H_aromatic_), 7.20 (d, *J* = 8.7 Hz, 4H, C–H_aromatic_), 6.44 (d, *J* = 17.3 Hz, 1H, CH_acrylate_), 6.14 (dd, *J* = 17.3, 10.4 Hz, 1H, CH_acrylate_), 5.87 (d, *J* = 11.8 Hz, 1H, CH_acrylate_), 4.99 (m, 1H, CH_2_–CH-CH_2_), 4.90 (m, 1H, NH) 4.28–4.18 (m, 2H, CH_2_), 3.52–3.41 (m, 2H, CH_2_), 3.21 (m, 4H, 2 CH_2_).

^13^C NMR (101 MHz, CD_2_Cl_2_, 25 °C): δ 165.8 (CO), 155.3 (CO), 134.8 (C_aromatic_), 132.0 (CH_aromatic_), 131.1 (CH_aromatic_),128.0 (CH_acrylate_), 120.2 (C_aromatic_), 71.9 (CH), 63.3 (CH_2_), 40.1­(CH_2_), 36.5 (CH_2_).

*m*/*z*: (Li+): 581.94 (100.0%), 582.94 (25.3%), 583.94 (56.6%), 584.94 (11%), 585.94 (2.1%), 586.9 (1.1%).

#### 2.1.3 Synthesis of Iodo Phenyl Urethane Acrylate, IPhUA

IPhUA was synthesized by reacting the respective alcohols with isocyanato ethyl acrylate described above in DMSO (Scheme 1C). The choice of solvent for the reaction of IEA with the iodo alcohol used in this study was due to the insolubility of the starting material iodo alcohol in most common solvents like dichloromethane (DCM), tetrahydrofuran (THF), and acetone. The reaction was carried out by stirring the reactants at room temperature for 4 h using dibutyltin dilaurate (DBTDL) as the catalyst. Products were obtained in >90% yield as white powder solids. FTIR, ^1^H NMR, ^13^C NMR, and mass spectroscopy confirmed its structure (see Supporting Information).

FTIR (cm^–1^): 3241 (N–H); 3041 (C–H); 2939–2859 (C–H); 1720 (C=O); 1635, 1618 (C=C); 693 (C–I).

^1^H NMR (400 MHz, CDCl_3_, 25 °C): δ 7.68–7.57 (m, 2H, CH_aromatic_), 7.19 (d, *J* = 8.3 Hz, 2H, CH_aromatic_), 6.69 (br-s, 1H, NH), 6.48 (dd, *J* = 17.3, 1.4 Hz, 1H, CH_acrylate_), 6.18 (dd, *J* = 17.3, 10.4 Hz, 1H, CH_acrylate_), 5.90 (dd, *J* = 10.5, 1.4 Hz, 1H, CH_acrylate_), 4.44 (s, 4H, 2 CH_2_).

^13^C NMR (101 MHz, CD_2_Cl_2_, 25 °C): δ 165.8 (CO) 152.8 (CO), 137.9 (C_aromatic_), 137.8 (CH_aromatic_), 131.1­(CH_acrylate_), 127.9 (CH_acrylate_), 120.5 (C_aromatic_), 86.1­(C_aromatic_), 63.1­(CH_2_), 62.4 (CH_2_).

*m*/*z*: (Li+): 367.99 (100.0%), 368.99 (13.3%), 370.00 (1.6%).

#### 2.1.4 Nuclear Magnetic Resonance

NMR spectra were recorded on a Bruker AVANCE-III 400 NMR spectrometer at 25 °C in deuterated solvents. All chemical shifts are reported in parts per million relative to the deuterated solvent used.

### 2.2 Hansen Solubility Parameters

Hansen Solubility Parameters in Practice (HSPiP) software was used to predict the influence of the HRI monomer structure and composition on solubility within polyurethane. The Hansen solubility parameters, δ_*d*_,δ_*p*_, andδ_*h*_, were found by entering the simplified molecular-input line-entry system (SMILES) representative of each studied molecule and calculated using a group-contribution method.^33^

### 2.3 Film Preparation

2-stage films were prepared in a 250 μm x 10 mm × 30 mm geometry by premixing the corresponding amount of the writing monomer (in weight percentage relative to the entire formulation) with a 1 wt % TPO photoinitiator (in weight percentage relative to the monomer loading) and the difunctional polyol (polyol 2000) in a 20 mL vial containing a minimum amount of acetone (used to solubilize the solid photoinitiator); the mixture was vortexed until homogeneous, and then a stoichiometric amount (OH/NCO 1:1) of Desmodur N3900 trifunctional isocyanate was added to the vial and vortexed again. Air bubbles and the remaining solvent were then removed by vacuum. The resin mixture was sandwiched between clean 1 cm × 3 cm glass slides using binder clips with a polyethylene terephthalate spacer of 250 μm thickness. Samples were placed in an oven at 60 °C overnight to ensure a full isocyanate/alcohol reaction. This was verified by analyzing the disappearance of the NCO peak (∼2200 cm^–1^) on a film sample using FTIR spectroscopy.

### 2.4 Real-Time Fourier Transformed Infrared

Due to the nature of the neat HRI monomers studied (i.e., BPTPUA is a liquid, whereas Br-BPTPUA and IPhUA are opaque powders), the only method of characterizing polymerization kinetics was to formulate the monomers in a 2-stage material scheme. Additionally, because the targeted applications largely utilize 2-stage materials, characterization of neat monomer kinetics would not be relevant. Polymerization kinetics of HRI monomers swollen in polyurethane were analyzed using a Nicolet 8700 FTIR spectrometer in transmission mode with a horizontal sample holder designed to irradiate the sample during real-time measurements, as reported elsewhere.^34^ Irradiation was performed using a UV light source (EFOS Acticure A4000 with 365 nm bandpass filter) at intensities ranging from 10 to 80 mW/cm^2^for 4 min. Real-time normalized monomer concentration [*M*(*t*)] was found by integrating the area under the 6170 cm^–1^ carbon double bond peak using a series scan during photopolymerization, integrating over the range of 6223–6136 cm^–1^ with a sampling frequency of 3.8 Hz. Normalized monomer concentration was calculated with eq 4, where *A*_*C*=*C*_(*t*) is the integrated area under the 6170 cm^–1^ peak at time *t* and *A*_*C*=*C*_(0) is the area when *t* = 0, the initial peak area representing the initial monomer concentration [*M*(0)]. A set of rate equations, discussed later, were fit to normalized monomer concentration [*M*(*t*)] and resulted in smooth [*M*(*t*)] curves that represented the FTIR data to within *R* ^2^ ≥ 0.95. The rate of polymerization *R*_p_ was calculated by taking the negative derivative of the solution to the monomer rate equation with respect to time (eq 5).

4 $$\left[M\left(t\right)\right]=\frac{A_{C=C}\left(t\right)}{A_{C=C}\left(0\right)}$$

5 $$R_{p}=-\frac{d\left[M\left(t\right)\right]}{dt}$$

### 2.5 Flood Cure Procedure

In this work, the terms “flood cure” and “post cure” refer to the exposure of 2-stage films to an incoherent light source (EFOS Acticure A4000 with 365 nm bandpass filter) until a sufficient optical dose was absorbed to reach the maximum possible conversion, verified by the disappearance of the 6170 cm^–1^ acrylate peak in FTIR. All subsequent mentions of “flood cure” and “post cure” refer to this method and were performed using the same equipment/procedure.

### 2.6 Refractive Index Measurements

HRI monomers were characterized in 2-stage films because the fundamental material properties (refractive index, rate of polymerization, shrinkage) change once swollen in a rubbery host matrix. The refractive index of 2-stage films (HRI monomers swollen in a polyurethane network) was measured five times per wavelength using a Metricon 2010/M prism coupler in TM mode at 405, 525, 637, 986, and 1546 nm at 30 °C before and after optical flood cure of the HRI monomers. The change in refractive index versus temperature was measured at 637 nm from 30 to 70 °C by varying the temperature of the prism and coupling head on the Metricon 2010/M prism coupler for both pre- and post-cure 2-stage films. Three thermal cycles were performed to generate statistics and determine the thermal stability. The refractive indices of neat liquid BPTPUA monomer samples were measured using an Abbe refractometer at room temperature at the sodium-d line (589.3 nm). The native states of Br-BPTPUA and IPhUA monomers are white powders, which made direct neat monomer refractive index measurements impossible.

### 2.7 Dynamic Mechanical Analysis

A TI Instruments RSA-G2 instrument was used for dynamic mechanical analysis (DMA). DMA was performed on 1-stage (polyurethane) and 2-stage films (250 μm × 10 mm × 20 mm) and before and after an optical flood cure (EFOS Acticure A4000 with 365 nm bandpass filter). The films were monitored at a frequency of 1 Hz while heated from −80 to 60 °C at a rate of 3 °C/min under an oscillatory strain of 0.03%. The glass transition temperature (*T*_g_) was determined as the peak of the tan­(δ) curve.

### 2.8 UV–Vis Spectroscopy

Broadband transmission measurements were performed using a Thermo Scientific Evolution 300 UV–vis spectrometer on 250 μm thick 15 wt % 2-stage films sandwiched between 1 mm glass slides before and after flood cure. Transmission data was recorded using a xenon lamp over the 350–1100 nm range at 1 nm resolution with a sampling rate of 240 nm/min. 100% transmission and 0% transmission baselines were initially performed and applied to all following measurements. HRI monomer conversion to the polymer was achieved by an optical flood cure. Polyurethane (1st stage) was included for completeness.

### 2.9 Transparency and Haze

Transparency and haze of 2-stage thin films and 1st-stage films (polyurethane) were characterized pre- and post-optical flood cure using a haze meter (BYK Instruments haze-gard (i) in accordance with the ASTM D1003-21 standard). Statistics were generated by taking five measurements at randomly chosen film locations.

### 2.10 Microlithography and Resolution Characterization

Laser micropatterning was performed using a Chameleon TI/Sapphire (Coherent) laser tuned to 730 nm for multiphoton patterning mounted on a Zeiss LSM 710 confocal microscope equipped with a 10*X*/0.5NA air objective (Item no.: 440135-0000-000), according to a previously described method.^35^ Digital micropattern masks (custom.ovl files) were prepared using MATLAB. All patterns were created with a pixel size of 0.42 μm^2^, a pixel dwell time of 0.79 μs, and 8 scans. Imaging and patterning were performed using the same objective. Resolution (the smallest negative feature that can be patterned) and critical dimension (smallest positive feature) of the 2-stage films were quantified by fitting peaks to a line plot of the triplicate-averaged intensity modulation along the X-direction (Figure S20). The smallest distance between identifiable positive and negative intensity modulated peaks in the positive feature section of the resolution pattern is the smallest positive feature that can be patterned (critical dimension), and the smallest distance between two peaks in the negative feature section of the pattern is the smallest negative feature that can be patterned (resolution). See Supporting Information for further discussion.

### 2.11 Holography

Volumetric holographic gratings were recorded in 2-stage films cast between 1 mm thick glass slides separated by 25 μm spacers exposed to interference of two coherent plane-waves produced by a narrowband λ = 405 nm laser diode (Coherent OBIS 405 nm LX 40 mW SF) (Figure S20). The power-matched s-polarized beams intersected the recording plane at an internal half angle of 12.5°, producing a grating with period Λ = 622 nm. Carbon-doped PDMS was laminated onto the back of the glass/2-stage film/glass package to minimize unwanted back reflections during recording.^36^ The samples were irradiated with an average intensity of 5 mW/cm^2^ for 30 s. After recording, the samples were rotated with respect to the Bragg-matched probe beam (λ = 637 nm, 1 mm diameter Thorlabs PL202) with power meters (Newport 2936-R and 918D-UV *x*2) collecting the transmitted and diffracted light, respectively, without initiating polymerization. Diffraction efficiency η was calculated as the ratio of diffracted light power to the sum of the transmitted light power. This efficiency as a function of sample rotation angle θ was fit to coupled wave theory (CWT), established by Kogelnik^37^ and implemented by Uchida.^38^ The fit to the CWT resulted in estimations of the diffraction grating film thickness L and the refractive index modulation *n*_1_. After characterization of the angular selectivity, the diffraction gratings were flood cured for 15 min at 1.5 mW/cm^2^with a 405 nm LED (Thorlabs SOLIS-405C) to consume the unreacted TPO/monomer and stabilize the index structures.

## 3 Results and Discussion

### 3.1 Refractive Index

The concentration of the 2nd-stage monomer is a fundamental design choice in 2-stage materials, with increasing concentration leading to a higher dynamic range Δ*n* but also more shrinkage σ and potential for phase separation, leading to haze generation. The refractive index of 2-stage films at various monomer loading pre- and post-optical flood cure (Figure 2) illustrates the dynamic range of these 2-stage materials and, thus, the tunability of the films to achieve precise optical properties. 2-stage BPTPUA (the control monomer) had a lower refractive index (Table 1) than both the brominated version Br-BPTPUA and the iodized variation IPhUA, which validates reported monomer design via inclusion of halogenated pendent groups. Despite the halogenated monomers exhibiting the highest pre- and post-cure refractive index, all 2-stage films had comparable dynamic ranges at the same monomer loading, implying that the pendent halogens increase the baseline refractive index but have no effect on the change upon polymerization. Furthermore, it was found that the maximal weight percent (wt %) loadings of Br-BPTPUA and IPhUA, e.g., 30 wt % and 15 wt %, respectively, were significantly lower than that of nonhalogenated BPTPUA, which could achieve up to 40 wt % before additional monomer would not dissolve in formulation. This is a consequence of the pendent halogens reducing miscibility within the polyurethane host, quantified by the large mismatch in the Hansen solubility parameter δ_*d*_ between polyurethane and Br-BPTPUA/IPhUA (Table S1), and clearly demonstrates the previously discussed trade-off between the refractive index and miscibility in monomer design for 2-stage material applications.

**Figure 2:**
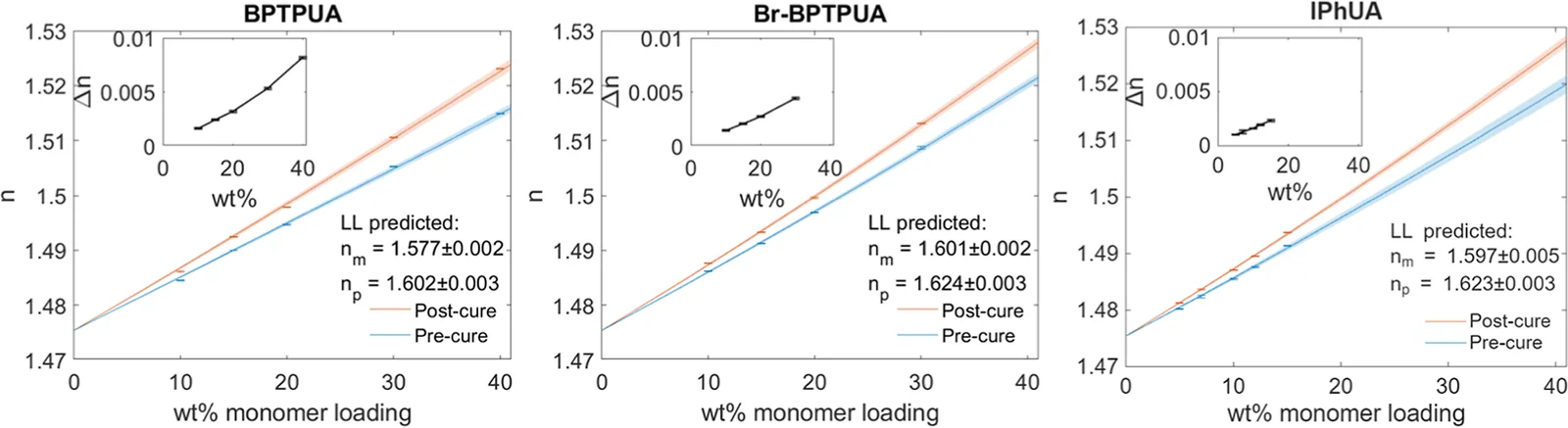
Refractive index at 637 nm versus monomer loading fit to the Lorentz–Lorenz (LL) relation (eq 1) to estimate the expected neat monomer *n*_m_ and polymer *n*_p_ index. The shaded regions represent the upper and lower 95% confidence bounds of the expected 2-stage refractive index using the extrapolated neat monomer/polymer index. The inset plot represents the dynamic range Δ*n*, i.e., the difference in pre vs post cure.

**Table 1:** Refractive Index Properties of 2-Stage 15 wt % HRI Monomer Films Pre Cure and Post Cure, and the Difference Δ*n*, Measured at λ = 637*nm* ^a^

	BPTPUA	Br-BPTPUA	IPhUA
pre cure *n*	1.4900	1.4912	1.4914
post cure *n*	1.4924	1.4932	1.4937
Δ*n*	0.0024	0.0020	0.0023
*n*_p,fit_	1.6018	1.6241	1.6232
*n*_p,σ_	1.6181	1.6368	1.6320

a Comparisons of the 2-stage index vs wt % loading fit polymer index *n*_p,fit_ and the upper bound of the polymer index *n*_p,σ_ assuming unimpeded shrinkage and no change to polarizability.

The Lorentz–Lorenz relation (eq 1) was used to fit to the refractive index vs wt % loading of 2-stage films for the three monomers and resulted in estimates of the neat monomer *n*_m,fit_ and polymer index *n*_p,fit_ (see Supporting Information for discussion). The upper and lower 95% confidence intervals of the estimated neat indexes were then used to generate upper and lower bounds on the expected refractive index measurements of 2-stage films (i.e., the shaded regions of the Figure 2 plots).

The Lorentz–Lorenz regressions were internally consistent and found that with no HRI monomer loading, (e.g., wt % = 0), the expected refractive index was that of the polyurethane, *n* = 1.4775 ± 0.0011. Because Br-BPTPUA and IPhUA monomers are opaque powders and thus cannot be characterized in a pure form, fits to the refractive index of 2-stage films with variable monomer loading were the only method of quantifying the monomer index. This is similar to methods used to characterize nonliquid monomers,^39,40^ but in this case the solvent is a solid host and thus imparts additional constraints on polymer shrinkage, making the results relevant to the intended 2-stage material applications. Critically, the fits for the monomer and polymer index were performed independently using pre- and post-cure 2-stage measurements, respectively, and assumed shrinkage occurred with no resulting stress on polyurethane (e.g., polymerization-induced shrinkage was not impeded by the surrounding soft host matrix).

However, it is well-known that the conversion of acrylate double bonds to single bonds decreases polarizability, and vitrification prevents the full contraction of the monomer to a polymer; both effects decrease the maximum refractive index.^41^ These effects were not independently characterized, so the change in refractive index cannot be used to calculate the shrinkage that occurred. Thus, this predicted polymer index *n*_p,σ_ is an upper bound on the refractive index, assuming complete shrinkage, in the limit that there is negligible polarizability change, providing insight into the coupled role shrinkage and polarizability play in these 2-stage materials.

For the HRI presented, the effective polymer refractive index from fitting to 2-stage index vs wt % data, *n*_p,fit_, was significantly lower than the predicted polymer index assuming full shrinkage and unchanging polarizability, *n*_p,σ_ (Table 1). This comparison provides insight into the potential gains in a dynamic range that could be achieved by, e.g., decreasing the host modulus to enable more complete shrinkage or increasing the monomer molecular weight to reduce the change in polarizability caused by the reaction of the single double bond. While it was not possible to separate the effects of incomplete shrinkage and changing polarizability in this experimental setup, clearly, this discrepancy between measured *n*_p,fit_ and predicted *n*_p,σ_ motivates why it is critical to characterize these HRI monomers in the 2-stage material scheme relevant to the intended applications. For example, if the monomers were suspended in a solvent where shrinkage would be unimpeded by the soft host matrix, the change in refractive index upon polymerization Δ*n* would be significantly higher but not applicable to the intended 2-stage material applications that require a soft host matrix. This is a fundamental material design parameter because the neat refractive index of these monomers and polymers is not representative of their properties when formulated with polyurethane as a 2-stage material.

Refractive index measurements of 15 wt % HRI monomer loading 2-stage thin films (Table S2) and pure monomer/polymer (Table S3) were taken at various wavelengths spanning near UV (405 nm) up to near IR (1546 nm) (Figures S15A and S15B). Based on these measurements, two Abbe numbers (Tables S2 and S3) were generated. Both Abbe numbers presented here are measures of dispersion and are differentiated by the reference wavelength used; i.e., *V*_d_ references the Helium d line (588 nm) and *V*_e_ references the Mercury e line (546 nm).

2-stage thin films (15 wt % monomer swollen in polyurethane) were found to have typical Abbe numbers for polymers,^42^ with BPTPUA and its derivative Br-BPTPUA measured at 53 and 52 before optical flood cure, respectively. For context, polyurethane was found to also have an Abbe number of 53, which indicated that neither BPTPUA nor Br-BPTPUA monomers significantly altered the dispersive properties of the 1st-stage polyurethane. Notably, the introduction of the 2nd-stage IPhUA monomer lowered the 2-stage films’ Abbe number to 47, which implied that neat IPhUA likely exhibited strong dispersive properties. After flood cure, the IPhUA 2-stage films displayed increased dispersive properties, quantified by the drop in Abbe numbers to 42. Similarly, the BPTPUA and Br-BPTPUA 2-stage films showed an increase in dispersion once flood cured, decreasing both films’ Abbe numbers to 47. These results showed that the dispersion in polyurethane films swollen with BPTPUA or Br-BPTPUA was still dominated by the 1st-stage polyurethane prior to 2nd-stage curing, whereas the dispersive properties of post-cure films were dominated by the 2nd-stage HRI polymer. This was not true for IPhUA, where the 2nd-stage HRI species dominated the dispersive behavior before and after flood cure, as evidenced by the notably lower Abbe numbers pre and post cure.

To characterize the thermo-optic coefficient of the monomers, the change in refractive index vs temperature (*dn*/*dT*) was found for 2-stage films (Figure S15C,D). Because the refractive index depends on density, materials with positive coefficients of thermal expansion exhibit negative dn/dT’s.^43^

All formulations displayed a negative correlation between the refractive index and temperature (Figure S16A), validating that the 2-stage films behaved like typical rubbery polymers^44^ with positive coefficients of thermal expansion. This is further supported by the *dn*/*dT* measurements of pure 1st-stage polyurethane, BPTPUA monomer/polymer, and Br-BPTPUA polymer, which all reported negative thermo-optic coefficients (Figure S16B). Additionally, the thermo-optical coefficients remained largely unchanged over the temperature range and through multiple thermal cycles, indicating no notable thermally induced polymerization. By this method, the films were shown to be chemically stable up to 70 °C.

### 3.2 FTIR Photopolymerization Kinetics

Polymerization kinetics of 2-stage samples were collected at incident irradiation intensities ranging from 10 to 80 mW/cm^2^ with a 365 nm source. The normalized monomer concentration [M­(t)] exhibited a low signal-to-noise ratio (SNR) due to the low fractional concentration of the species being measured. Smoothing of the noisy data (i.e., a moving average) would have reduced the accuracy of extracted kinetic constants. Thus, the noisy data was fit with a kinetic model that directly incorporated the parameters of interest. The model, shown in eq 6, describes the time evolution of the three species controlled by five free parameters that was numerically integrated using MATLAB’s ode45 solver and fit to the collected FTIR data to generate representative monomer concentrations for each intensity. The materials model used was an inhibited photoinduced polymerization, where, as shown in the first equation, primary radicals [R·] are generated at a constant rate k_PI_I from photocleavage of TPO due to irradiation with intensity I and consumed via unchanging unimolecular termination with rate constant *k*_T1_, bimolecular termination with rate constant *k*_T2_, or reaction with oxygen [O_2_] in the resin at rate k_O2_. Oxygen is depleted by this reaction in the second equation. The third equation accounts for the monomer [M] reaction with a primary radical and the generation of the polymer at the rate constant *k*_p_. Diffusion of species into or out of the monitored region was not considered since entire films were uniformly exposed and sandwiched between glass slides, preventing in-diffusion of oxygen or additional monomer. To simplify the model, intensity was assumed to not vary through depth (see Supporting Information for further discussion on the optically thin approximation) and not vary with time (fitting only *t* ≥ *t*_light on_). Critically, the materials model does not account for vitrification, which is discussed later. Despite the noisy data, all fits of the model to the FTIR data achieved a goodness of fit of *R* ^2^ ≥ 0.95 (Figure 3A).

6 $$\frac{\partial \left[R\cdot \right]}{\partial t}=k_{PI}I-k_{T_{1}}\left[R\cdot \right]-{k_{T_{2}}{\left[R\cdot \right]}^{2}-k}_{o_{2}}\left[O_{2}\right]\left[R\cdot \right]$$

7 $$\frac{\partial \left[O_{2}\right]}{\partial t}=-k_{O_{2}}\left[R\cdot \right]\left[O_{2}\right]$$

8 $$\frac{\partial \left[M\right]}{\partial t}=-k_{p}\left[R\cdot \right]\left[M\right]$$

**Figure 3:**
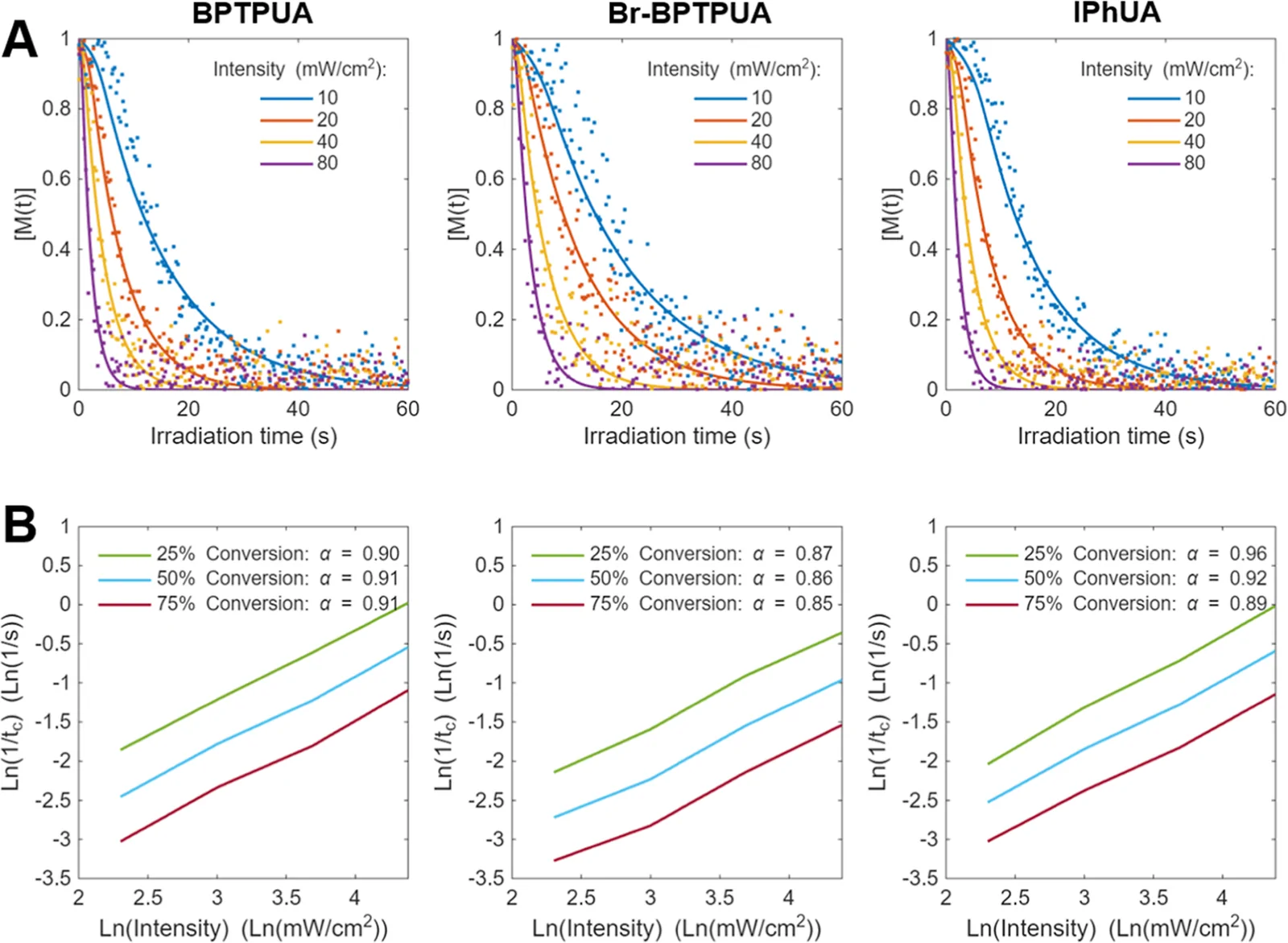
(A) Real-time FTIR data representing normalized monomer concentration plotted alongside optimized fits that represent collected data to within *R* ^2^ ≥ 0.95 for all monomers and intensities. Only the first 60 s after the light was initially turned on is shown. (B) The time to reach a specified conversion *t*_*c*_ for a range of intensities on a log–log plot to illustrate the scaling relationship between intensity and polymerization rate.

When irradiated with various light intensities, it was found that 2-stage films with 15 wt % HRI monomer loading reached effectively full conversion. Achievement of complete conversion occurs only in acrylate systems that do not vitrify, i.e., become glassy, at high conversions in the second stage.^29^ However, there are other reported^45^ 2-stage photopolymerizations utilizing acrylates swollen in polyurethane that readily achieve full conversion due to modest monomer loading. In these systems, the presence of the rubbery host helps to prevent vitrification, so the results and the assumptions made in the material model are reasonable for this specific material scheme. To note, this model would not be appropriate for photopolymerizations that do not reach full conversion due to the fact that the third equation does not support a final monomer concentration that is nonzero and the diffusion control of kinetics would lead to significant changes in the kinetic constants as a function of conversion.

An analysis of polymerization rate dependence on the initiating light intensity was then performed, similar to Scott et al.,^46^ with the goal of understanding the scaling behavior of these reactions. Briefly, the polymerization rate in free radical chain growth reactions is controlled by, among many factors, the dominant form of termination in the resin. In theory, typical acrylate polymerizations^29^ exhibit a half-power scaling law relationship between the polymerization rate and incident light intensity, *R*_p_ ∝ *I* ^0.5^, assuming that chain length-independent propagation and termination processes (combination or disproportionation) dominate, whereas other acrylate resins with reduced mobility have been reported^46^ to show a near-linear scaling of rate with intensity, *R*_p_ ∝ *I*, due to unimolecular termination dominating others that exhibit chain length-dependent reactions where the scaling exponent is between 0.5 and 1. Under the steady-state approximation, which assumes that the change in radical concentration is negligible compared to the reaction time, *R*_p_ is proportional to intensity *I*_0_ times the photoinitiator concentration [PI] as $R_{p}\propto {\left(I_{0}\left[PI\right]\right)}^{\alpha }$ . One way of determining an average rate to provide an indication of the scaling exponent, particularly when measuring conversion, is to use the time required to reach a certain conversion. That time, t_c_, is then inversely proportional to the average rate over the range of conversions. As such, here, the relationship is redefined as $\frac{1}{t_{c}}\propto {\left(I_{0}\left[PI\right]\right)}^{\alpha }$ , where *t*_*c*_ represents the time to reach a specific fractional conversion. By taking the log of both sides of the power law and performing multiple polymerizations at various intensities, a line was fit, and the exponent α was determined for all three HRI monomers (Figure 3B). The scaling between intensity and time to reach specific fractional conversions was significantly higher than expected for free radical chain growth reactions with chain length-independent kinetics (where α→0.5), with the results here indicating α→(0.8–0.9) for the monitored 2-stage reactions. This behavior indicated that these acrylate polymerizations exhibit chain length-dependent kinetics or potentially unimolecular termination in these 2-stage films. A near-linear scaling of rate with the initiation rate benefits applications such as holography because the polymer formed is proportional to dose, whereas sublinear polymerization rates generate unwanted harmonics in holograms. In direct-write lithography, sublinear scaling increases feature size and accumulation of unwanted polymerization out of the focal volume.^47^

### 3.3 Dynamic Mechanical Analysis

DMA of the 2-stage films and 1st-stage polyurethane was performed with the goal of characterizing how the different monomer structures affected the bulk glass transition temperature *T*_g_ and the storage modulus pre and post optical flood cure (Figure S17). It was found that all formulations produced 2-stage films with *T*_g_’s significantly below room temperature, and these *T*_g_’s increased a negligible amount upon photopolymerization (Table S4). As expected, polyurethane (compromising the bulk of the films) dominated the mechanical behavior of the 2-stage materials with a *T*_g_ of −55 °C, well below room temperature. This is a critical material design parameter in the 2-stage system since mobility and thus rate of polymerization are dramatically higher in 2-stage films that are flexible and rubbery compared to films that are stiff and glassy.^29^ Addition of the 2nd-stage HRI monomers slightly increased the *T*_g_, but the composite material remained well below room temperature. It is worth noting that all 2-stage films displayed similar *T*_g_’s pre and post cure, implying that monomer mobility should be similar across the samples. This is reasonable due to the low loading of monofunctional acrylates and the lack of covalent bonding to the rubbery host.

While there was a small but positive shift in *T*_g_ upon photopolymerization, the shape of the δ-tan curve changed substantially. The breadth of a tan­(δ) peak is often used as a proxy for the homogeneity of the material, i.e., more homogeneous materials have tall, narrow peaks, while heterogeneous materials have short, wide peaks.^48^ For the HRI monomers swollen in polyurethane, the tan­(δ) peaks consistently became shorter and wider, as evidenced by the full width at half-maximum (fwhm) increase, indicating increased inhomogeneity of the films post optical flood cure. UV–vis and haze measurements (Figure 4) also support the hypothesis that photopolymerization increases inhomogeneity in these films since transmission decreased and haze increased upon flood cure, possibly due to polymerization-induced phase separation (PIPS). However, chain growth monomers generally broaden in molecular weight distribution when polymerized,^29^ so broadening of the tan­(δ) peak cannot be solely attributed to PIPS. Films with greater wt % loading (Figure S18) were also characterized and exhibited an increased broadening of the tan­(δ) peak compared to the lower wt % loading films. Critically, the *T*_g_^′^s of higher wt % films remained well below room temperature.

**Figure 4:**
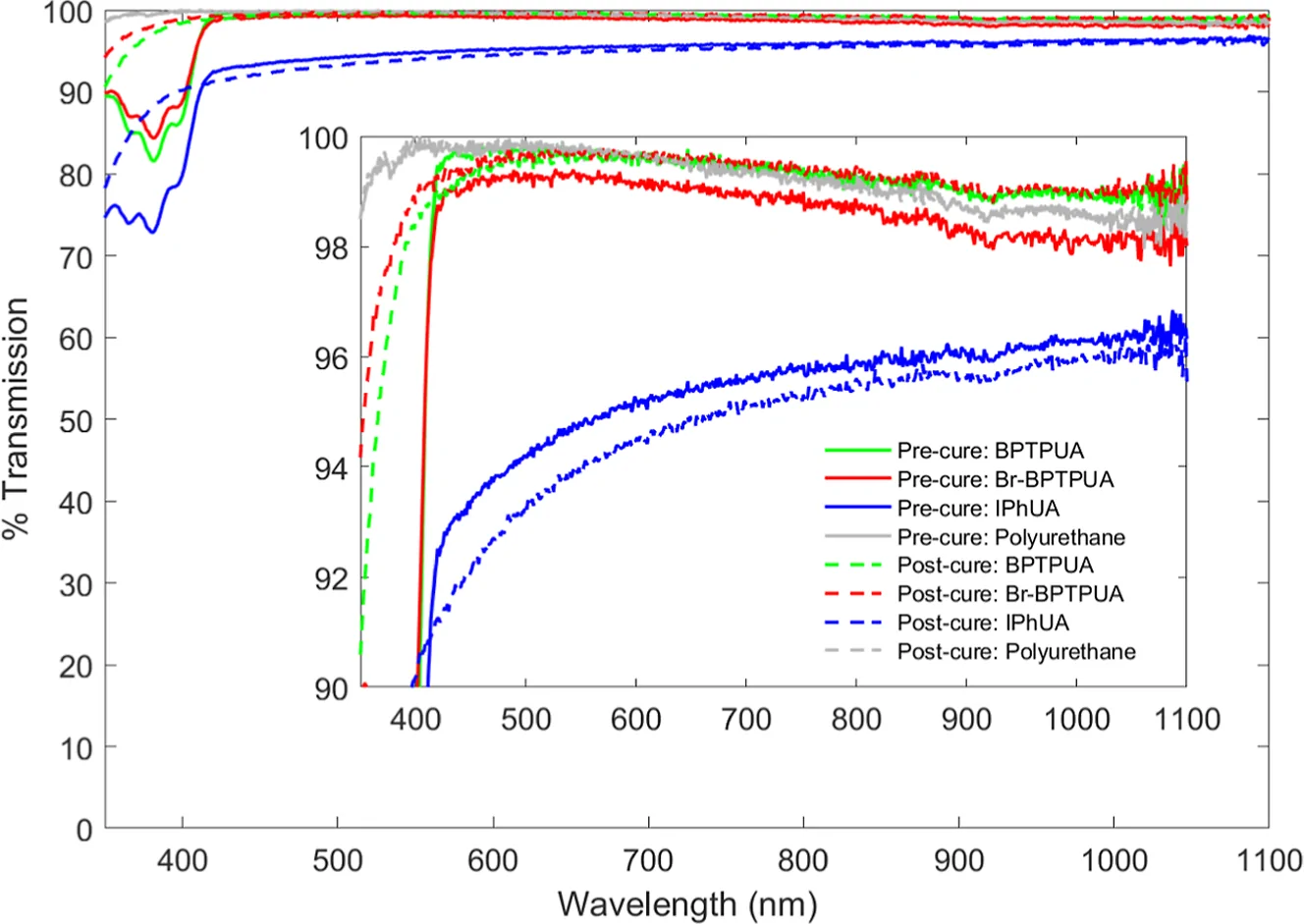
Broadband transmission data from 1st-stage polyurethane and 2-stage thin films (250 μm) between glass slides (1 mm).

### 3.4 UV–Vis Transmission

Broadband optical transmission data for the 1st-stage polyurethane and 2-stage 15 wt % HRI monomer films pre and post optical flood cure were collected in the near UV (350 nm) to near IR (1100 nm) (Figure 4). Pure polyurethane was included to highlight the effects of adding the 2nd-stage HRI monomers to the formulation and, as expected, exhibited a high baseline transmission that was unchanged pre and post flood cure. Due to the presence of TPO which is absorbed in the near UV range of 380425 nm,^49^ pre-cure transmission data was relatively low (*T* < 90%) for all monomers. Because TPO photo-bleaches, post-cure samples exhibited dramatically improved transmission at these low wavelengths. Outside of the TPO absorption spectra, transmission of BPTPUA pre and post flood cure was found to be essentially invariant, indicating no adverse effects (e.g., discoloration, haze) caused by photopolymerization. Conversely, transmission of Br-BPTPUA increased upon photopolymerization.

The IPhUA monomer pre and post optical flood cure exhibited the lowest transmission of all reported species, with the highest transmission occurring in the near IR range with a value of 95% and decreasing to ∼91%, approaching the TPO absorption spectra. The reduced transmission across all wavelengths was attributed to the lower solubility of the IPhUA monomer in polyurethane, causing partial demixing and resulting in increased scatter of transmitted light. This is likely a consequence of the relatively increased concentration of low solubility pendent halogens in IPhUA, evidenced by the increase to δ_*d*_ compared to Br-BPTPUA or BPTPUA. As IPhUA was irradiated, conversion of the IPhUA monomer to a polymer likely lowered the solubility of the species within polyurethane, causing PIPS and resulting in reduced transmission due to Rayleigh scattering. All changes in transmission were uniform across the visible spectrum for all three species in 2-stage films, implying that the C–Br and C–I bonds in the halogenated monomers were relatively stable under exposure conditions consistent with the targeted applications. Prolonged UV irradiation has the potential to degrade these bonds,^50^ creating ions and discoloration in the form of yellowing, but those exposure conditions are outside the scope of this work.

### 3.5 Transparency and Haze

Transparency and haze of the HRI monomers in polyurethane were quantified pre and post optical flood cure (Table S5). As expected, and in accordance with UV–vis transmission data, BPTPUA pre and post cure displayed the highest transparency and lowest haze of all the samples, which is expected because BPTPUA contains the urethane moiety that increases solubility within the urethane host matrix and does not contain pendant bromines or iodine that likely decrease solubility. 2-stage films containing Br-BPTPUA were also highly transparent with low haze, but notably, the haze decreased in post-cure Br-BPTPUA films. Regardless, both BPTPUA and Br-BPTPUA 2-stage films exhibited high transparency and low haze. IPhUA was demonstrably less transparent and had a higher baseline haze than the other formulations, likely due to the reduced solubility of the monomer within the polyurethane host matrix.

### 3.6 Two-Photon Direct Write Microlithography

All three 2-stage material schemes produced high-fidelity patterns, including microscale representations of their molecular structures (Figure 5A), resolution tests with submicron features (Figure 5B), and arbitrary shapes such as the CU Buffalo logo (Figure 5C). Analysis of the resolution tests revealed the critical dimension and resolution of the 2-stage films (Table S6). All films supported submicron features. BPTPUA and Br-BPTPUA exhibited relatively equal resolutions and critical dimensions, whereas the resolution for IPhUA was significantly higher than the critical dimension. Of note, imaging was performed with a 488 nm laser and a 0.5 NA objective, meaning that the microscope was only able to resolve features as small as 0.59 μm due to the diffraction limit (see Supporting Information). Thus, the quantified resolutions and critical dimensions of the films were at or slightly above the diffraction limit of the microscope.

**Figure 5:**
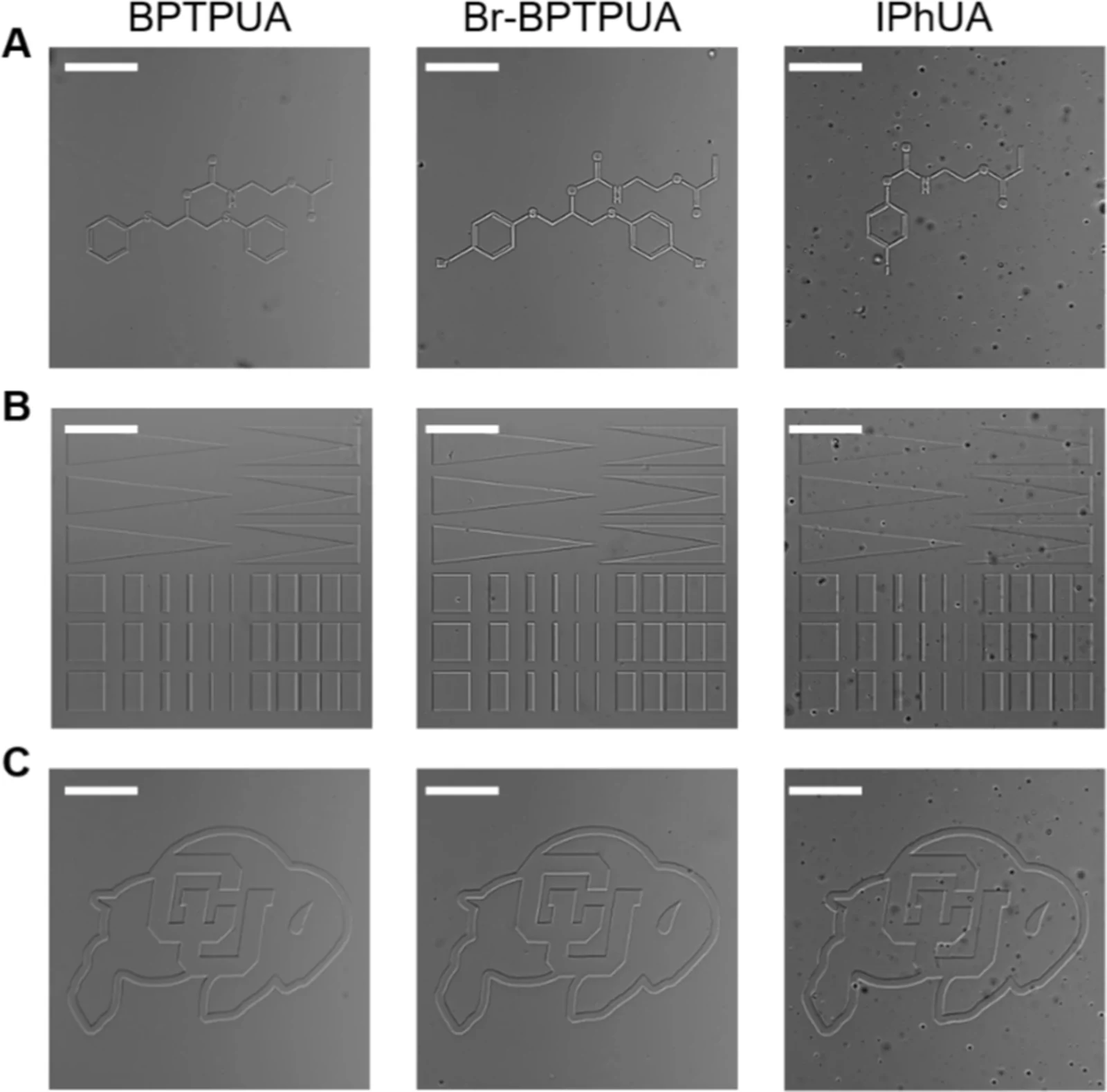
Two-photon (730 nm) microlithography of 15 wt % monomer loading 250 μm thick 2-stage films. Microscale chemical structures of writing monomers (A), resolution tests (B), and arbitrary patterns (C). Sparse insoluble aggregates are observed in the IPhUA samples, likely due to reduced solubility causing microphase separation. All scale bars = 100 μm. Images have been artificially brightened to enhance contrast.

### 3.7 Holography

Diffraction gratings with a pitch of Λ = 622 nm were recorded in 2-stage films containing the three HRI monomers at 15 wt % loading (Figure 6) and were characterized by rotating the samples with respect to a probe beam to determine diffraction efficiency as a function of angle η­(θ). By fitting coupled wave theory to η­(θ), the refractive index modulation *n*_1_ of the first harmonic, hologram thickness L, and peak diffraction efficiency η_max_ were determined for all three samples (Table 2). Note that the refractive index modulation *n*_1_ is an extrinsic property arising from the segregation of the acrylate and urethane species via diffusion and is thus not directly related to the intrinsic dynamic range Δ*n*. As expected from the previous section where submicrometer features were formed by direct write lithography, all HRI monomers produced high-fidelity diffraction gratings as evidenced by moderate (∼50%) to strong (∼70%) peak diffraction efficiency and Bragg selectivity curves η­(θ) that fit well to coupled mode theory. By this method, 2-stage films comprising these HRI monomers have been shown to produce high-quality diffraction gratings with submicron feature size.

**Figure 6:**
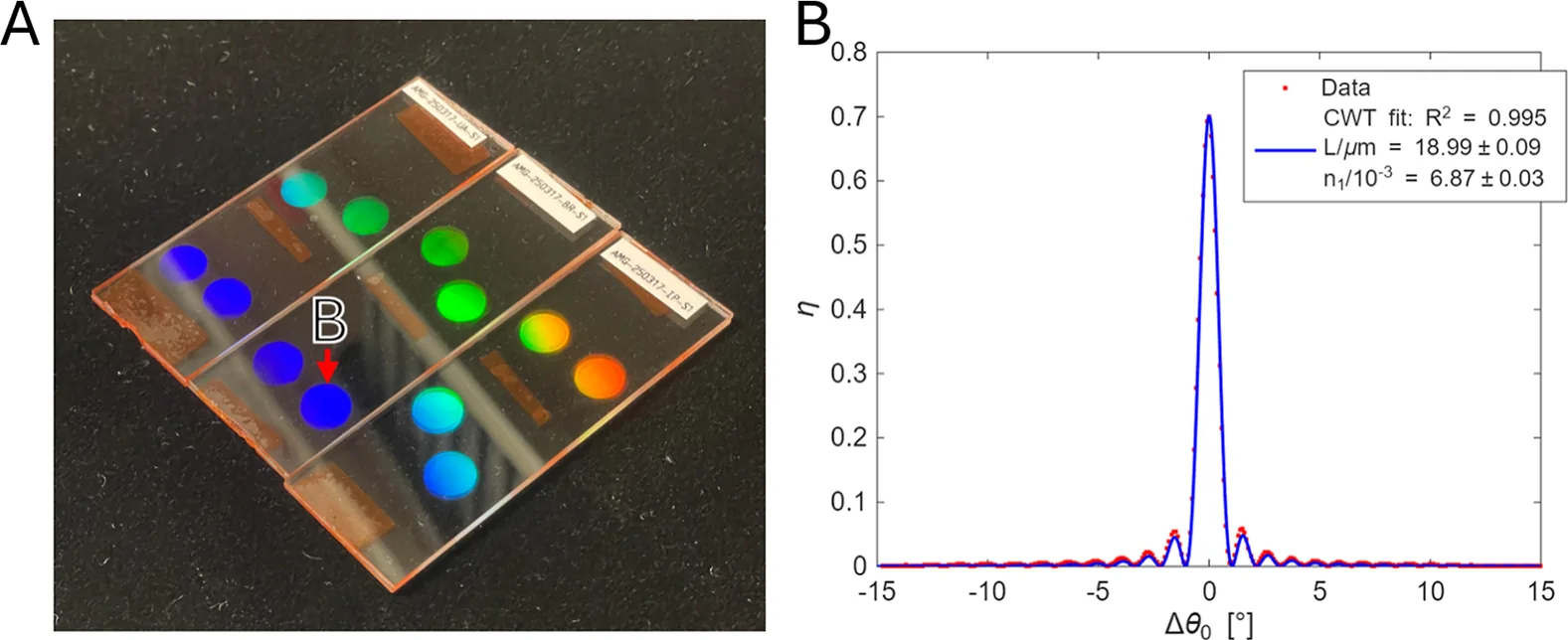
(A) Diffraction gratings recorded in 25 μm thick 15 wt % HRI monomer (Left to right: BPTPUA, Br-BPTPUA, IPhUA) 2-stage films and (B) the measured (red points) angular selectivity of a grating recorded in Br-BPTPUA swollen in polyurethane with the CWT fit (blue line).

**Table 2:** Results of CWT Fit (*R* ^2^ ≥ 0.995) to η­(θ) and Peak Diffraction Efficiency η_max_ for the Two New Halogenated HRI Monomers and BPTPUA at 15 wt % Loading in Polyurethane

grating property	BPTPUA	Br-BPTPUA	IPhUA
*n*_1_/10^–3^	6.69 ± 0.04	6.87 ± 0.03	6.11 ± 0.02
L (μm)	26.73 ± 0.14	18.99 ± 0.09	23.36 ± 0.08
η_max_(%)	71 ± 0.2	69.2 ± 0.2	47.8 ± 0.2

## 4 Conclusion

Two new HRI halogenated acrylate monomers designed for 2-stage optical material applications are presented and characterized when swollen in polyurethane. Notably, all characterization and resulting material properties (e.g., refractive index, shrinkage, kinetics, absorption, etc.) were only quantifiable and relevant when performed on 2-stage materials. Br-BPTPUA and IPhUA produced similar Δ*n* upon photopolymerization compared to BPTPUA but exhibited higher initial and final indices, a product of the pendent halogens. Consequently, the dispersion and haze of Br-BPTPUA and especially IPhUA increased compared to those of nonhalogenated BPTPUA. The photopolymerization process was characterized via real-time FTIR data fit to a three species kinetic model, which indicated that unimolecular termination processes dominate in these 2-stage films, making them excellent materials for applications such as holography. To show an application of these HRI monomers, 2-stage films were photopatterned with submicron resolution, and analysis via brightfield microscopy revealed successful refractive index modulation with submicron feature sizes. As further proof of concept, high-fidelity Bragg diffraction gratings with moderate to strong peak diffraction efficiency were photopatterned and characterized, validating that these 2-stage films support high-spatial-frequency structures with index modulation *n*_1_ on the order of 0.01.

## Supplementary Material



## Notes

Synthesis procedure and characterization of TIPhUA, NMR and mass spectrometry data, Hansen solubility parameter calculations, refractive index analysis and data, thin-film derivation, DMA results, transparency and haze methodology and data, spatial resolution procedure, and holography recording setup and results (PDF)
