# Supplementary material for: High-Refractive-Index Halogenated Urethane Acrylates in Polyurethane for 2‑Stage Material Applications
Source: ACS Appl Polym Mater. 2026 Mar 17;8(6):4220–32. doi: 10.1021/acsapm.5c04746 (PMC13730442; doi:10.1021/acsapm.5c04746)
Supplement: Supplementary file 1 [file ap5c04746_si_001.pdf]

Supporting information

High Refractive Index Halogenated Urethane Acrylates in Polyurethane for 2-Stage Material Applications

Arlo Marquez-Grap[†], Marianela Trujillo-Lemon[‡], Alexander J. Osterbaan[‡], Zachary R. Mora [‡], Andrew N. Sias[§], Bruce E. Kirkpatrick[‡], Kristi S. Anseth[‡], Christopher N. Bowman[†‡], and Robert R. McLeod[†§]*

[†]Materials Science and Engineering Program, University of Colorado Boulder, Boulder, CO 80303, USA

[‡]Department of Chemical and Biological Engineering, University of Colorado Boulder, Boulder, CO 80303, USA

[§]Department of Electrical, Computer, and Energy Engineering, University of Colorado Boulder, Boulder, CO 80309, UScA

Corresponding Author

*Email: Robert.Mcleod@colorado.edu

Table of Contents

1. **Synthesis of TIPhUA**
2. **NMRs of precursors and final products**
3. **Mass Spectroscopy**
4. **Solubility parameters**
5. **2-stage refractive index calculation**
6. **Refractive index measurements and dn/dt data tables**
7. **Optically thin calculation and materials model**
8. **DMA of increased loading films**
9. **Transparency and haze**
10. **Resolution test analysis**
11. **Holography**

1. Synthesis of TIPhUA

During the initial monomer design, we discovered that a tri-iodo variation of the IPhUA monomer could be synthesized, which we termed TIPhUA. However, we found that TIPhUA was not soluble in the polyurethane at any wt% loading, likely due to the increased concentration of elemental halogens on the monomer structure. Consequently, 2-stage films of TIPhUA could not be formed and the TIPhUA monomer was not characterized in this study. The synthesis for TIPhUA has been provided below for completeness.

Synthesis of 2,4,6-Triiodophenyl urethane acrylate, TIPhUA

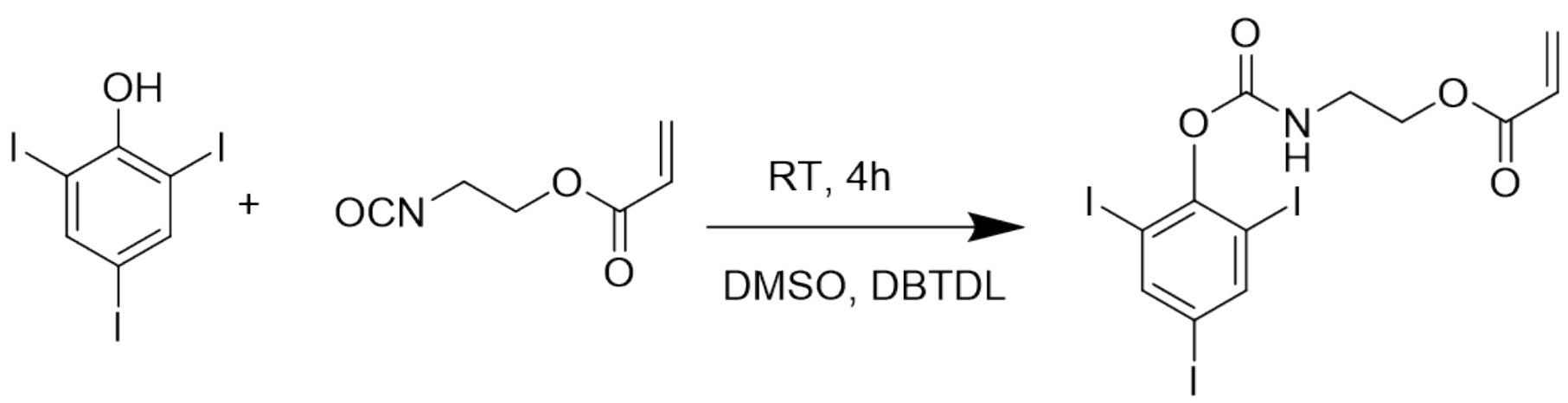

TIPhUA was synthesized by the reaction of the respective alcohols with isocyanato ethyl acrylate as described above in DMSO. The choice of solvent for the reaction of isocyanate ethyl acrylate with the two iodo alcohols used in this study was due to the insolubility of the iodo alcohols starting materials in most common solvents like dichloromethane (DCM), tetrahydrofuran (THF) and acetone. The reaction was carried out by stirring the reactants at room temperature for 4 h using dibutyltin dilaurate (DBTDL) as the catalyst. Product was obtained in > 90% yield as a white powder solid. ^{1}H NMR, ^{13}C NMR, and mass spectroscopy confirmed the structure.

^{1}H NMR (400 MHz, CD_2Cl_2) δ 8.15 (s, 2H, $CH_{aromatic}$), 6.47 (dd, J = 17.3, 1.4 Hz, 1H, ($CH_{acrylate}$), 6.28 – 6.16 (m, 1H, $CH_{acrylate}$), 5.91 (dd, J = 10.4, 1.4 Hz, 1H, ($CH_{acrylate}$), 5.69 (s, 1H, NH), 4.41 – 4.33 (m, 2H, CH_2), 3.64 (td, J = 5.9, 5.0 Hz, 2H, CH_2).
^{13}C NMR (101 MHz, CD_2Cl_2) δ 165.87 (CO), 151.59 (CO), 147.05 ($CH_{aromatic}$), 146.53 ($C_{aromatic}$), 131.24 ($CH_{acrylate}$), 128.03 ($CH_{acrylate}$), 92.18 ($C_{aromatic}$), 91.13 ($C_{aromatic}$), 63.11 (CH_2), 40.63 (CH_2).
m/z (Li+): 619.79 (100.0%), 620.79 (13.3%), 621.79 (0.5%).

2. NMRs of precursors and final products

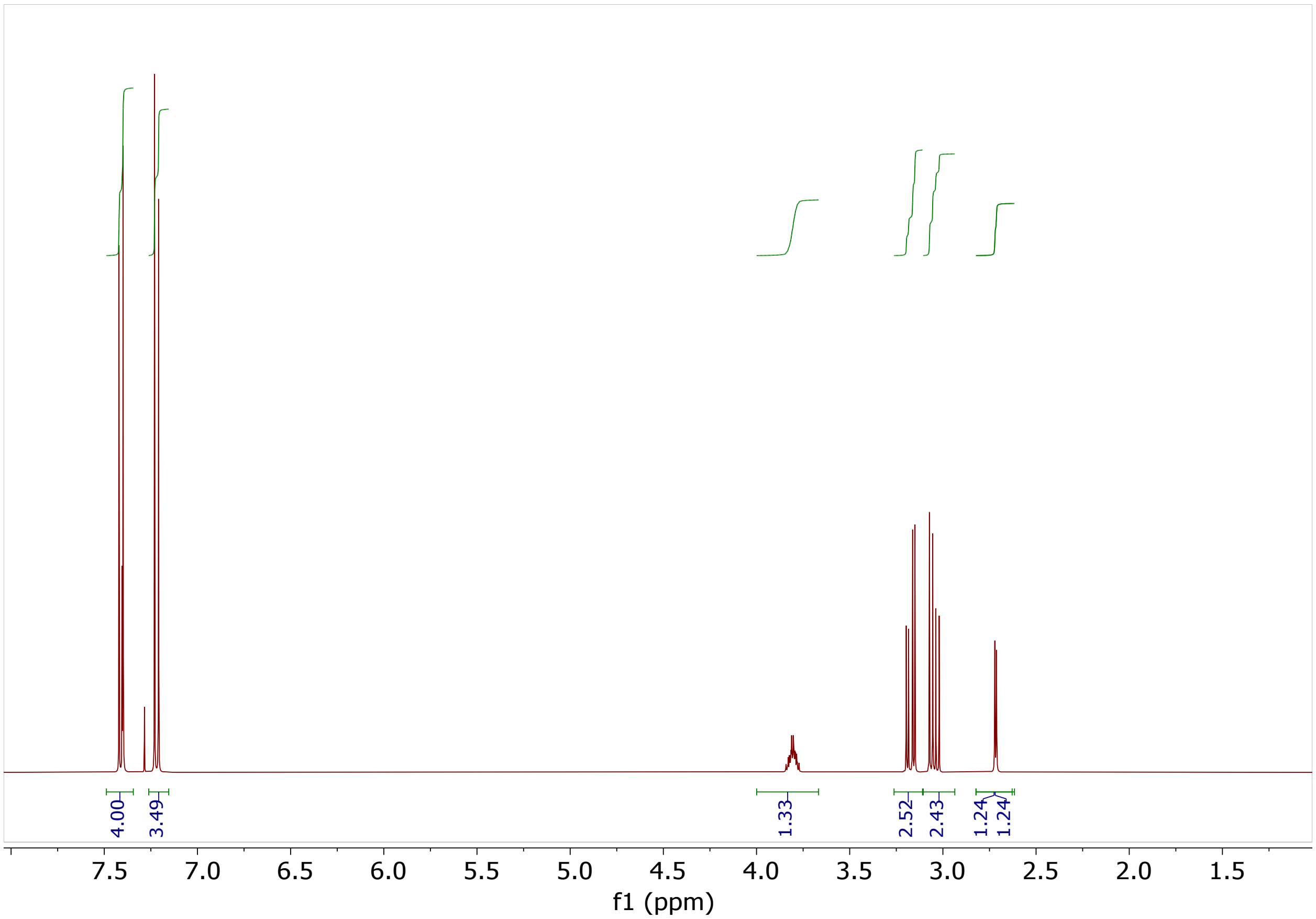

Figure S1: ^{1}H NMR of Br-BPTP

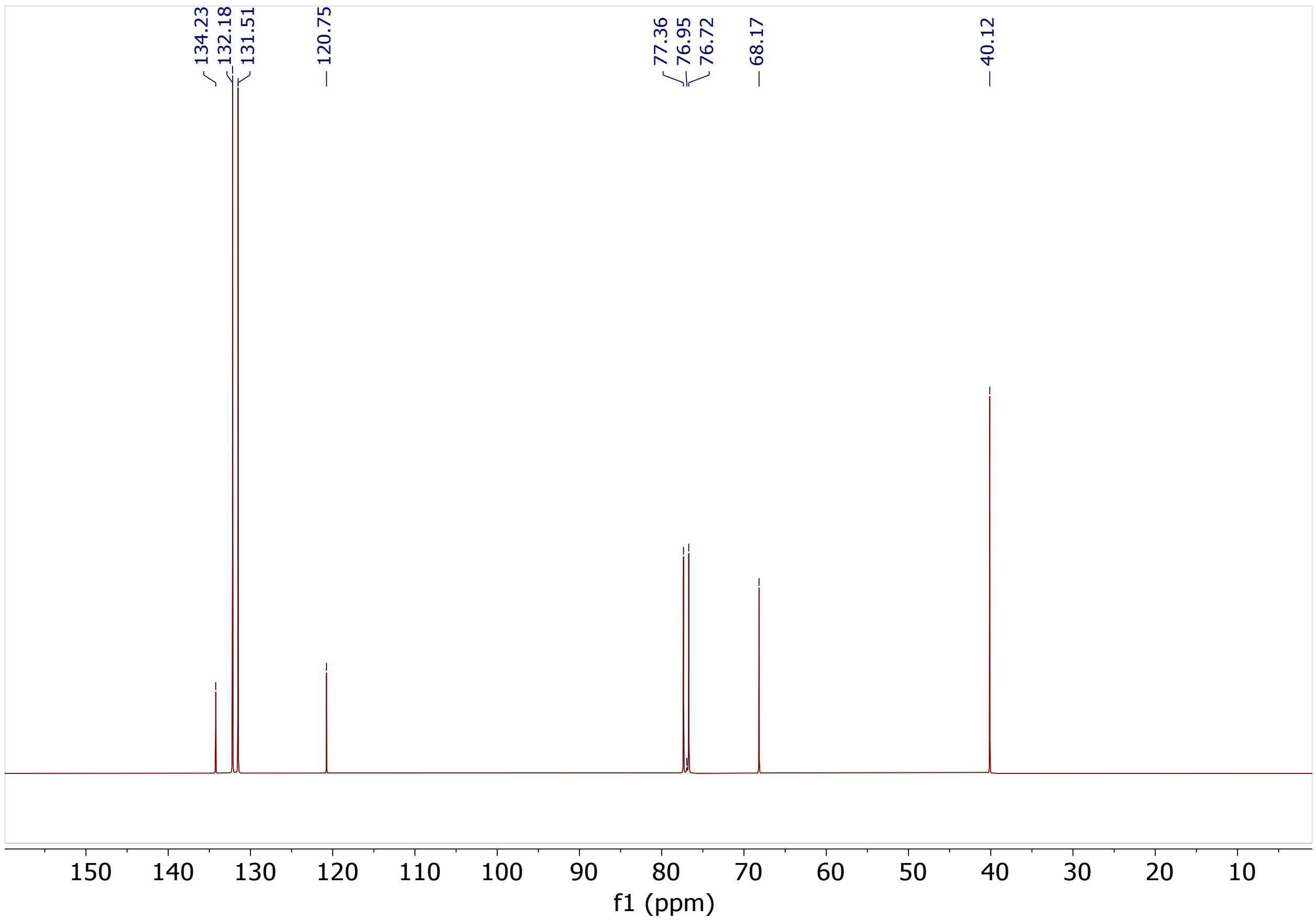

Figure S2: ^{13}C NMR of Br-BPTP

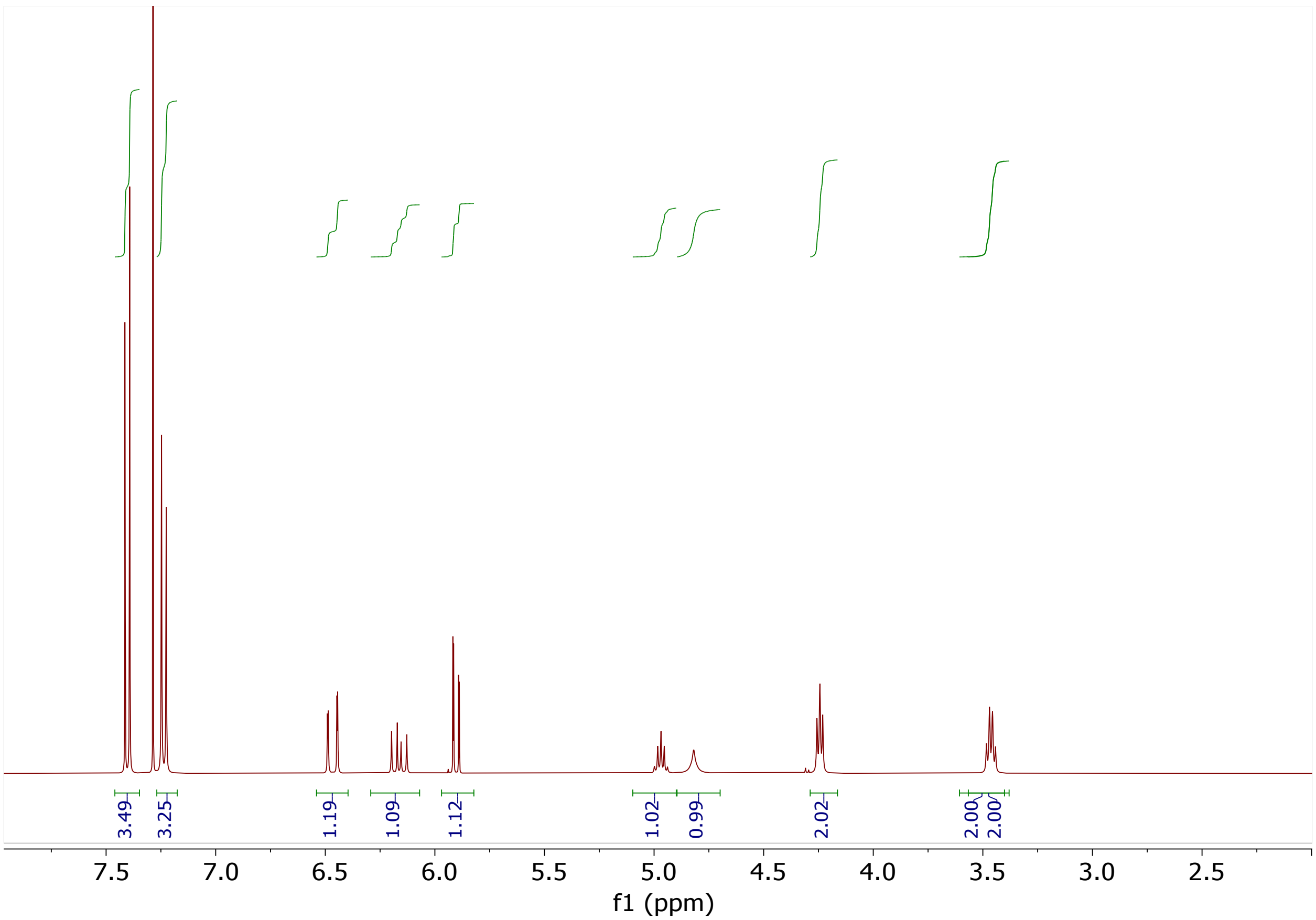

Figure S3: ^{1}H NMR of Br-BPTPUA

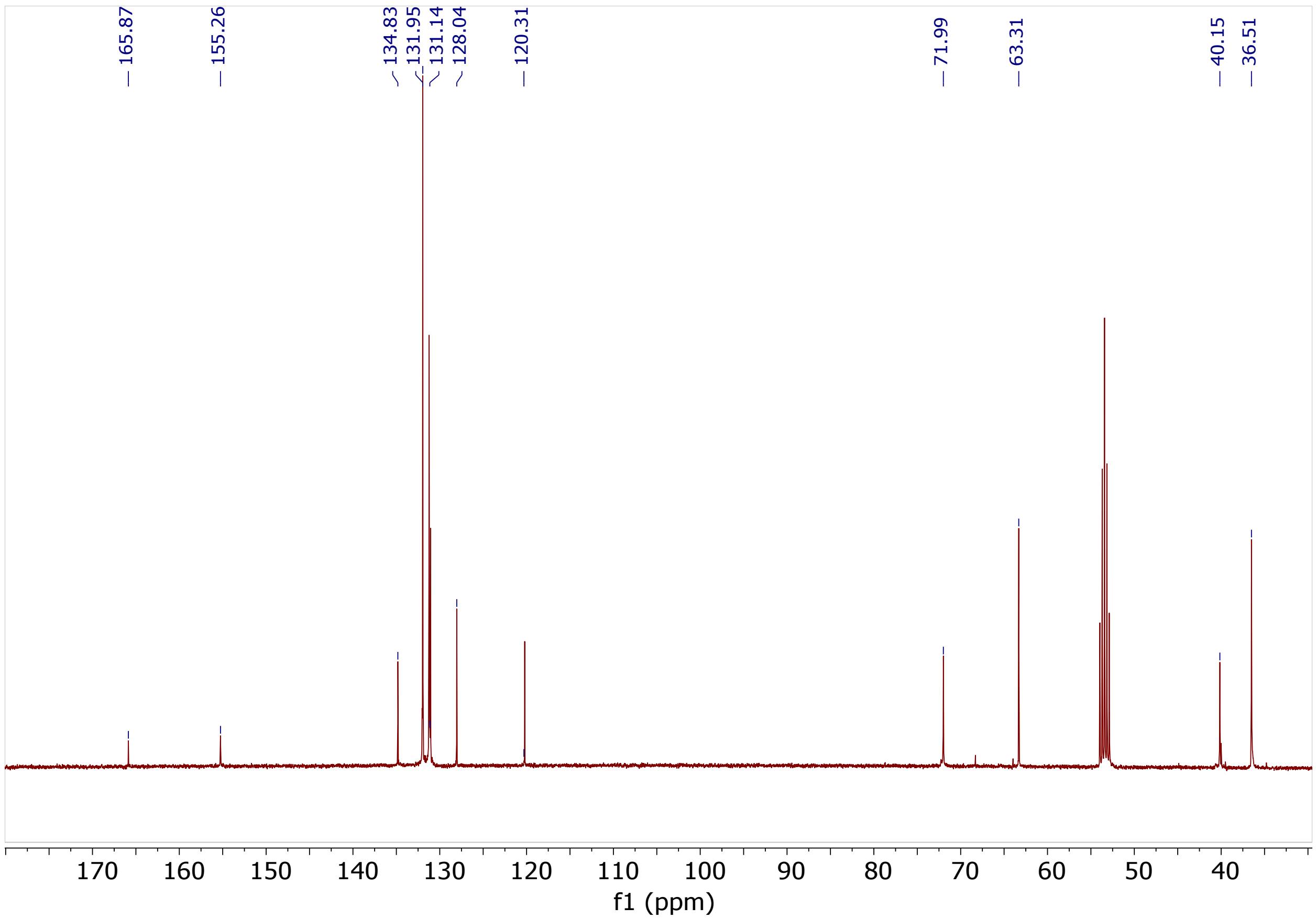

Figure S4: ^{13}C NMR of Br-BPTPUA

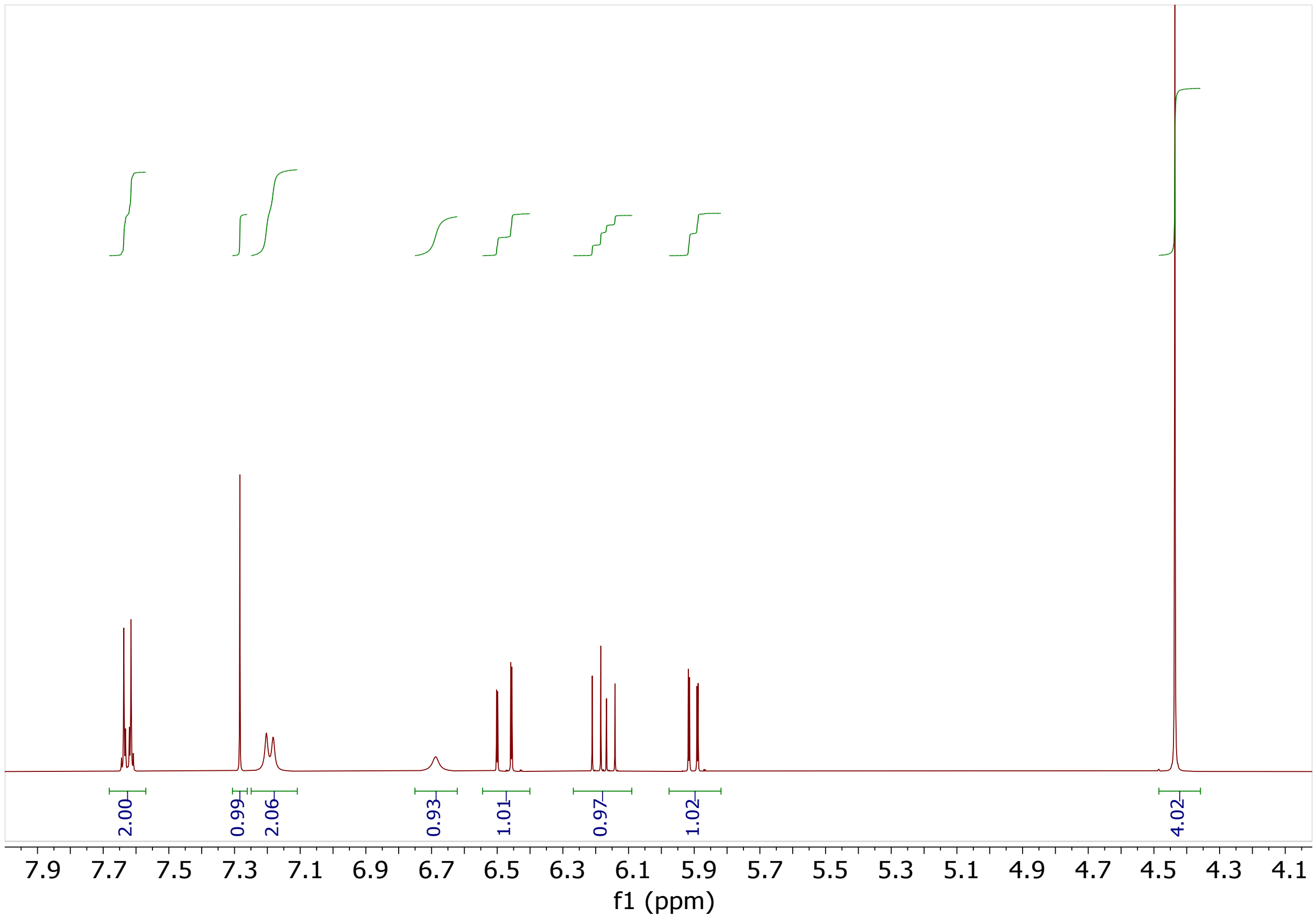

Figure S5: ^{1}H NMR of IPhUA

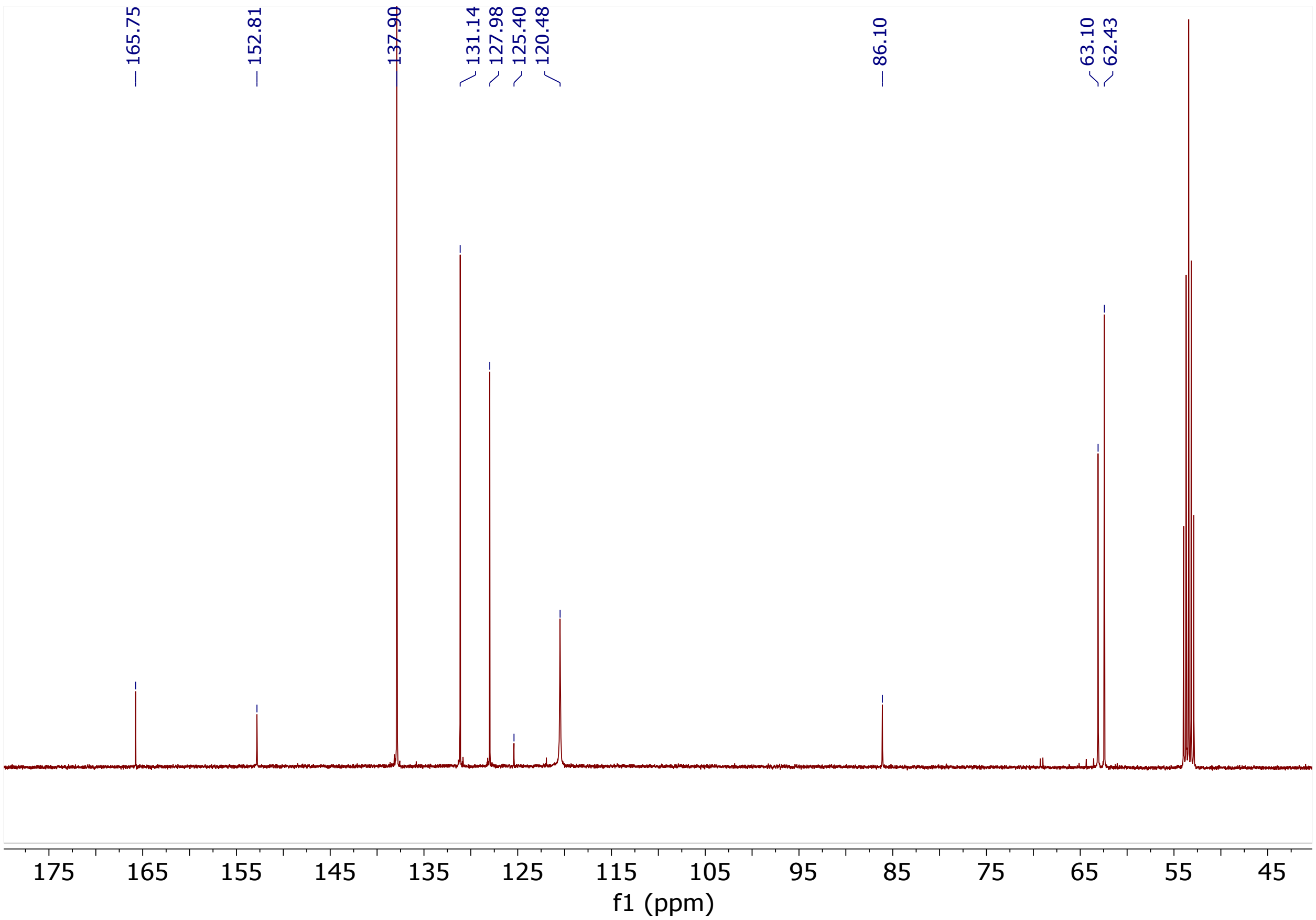

Figure S6: ^{13}C NMR of IPhUA

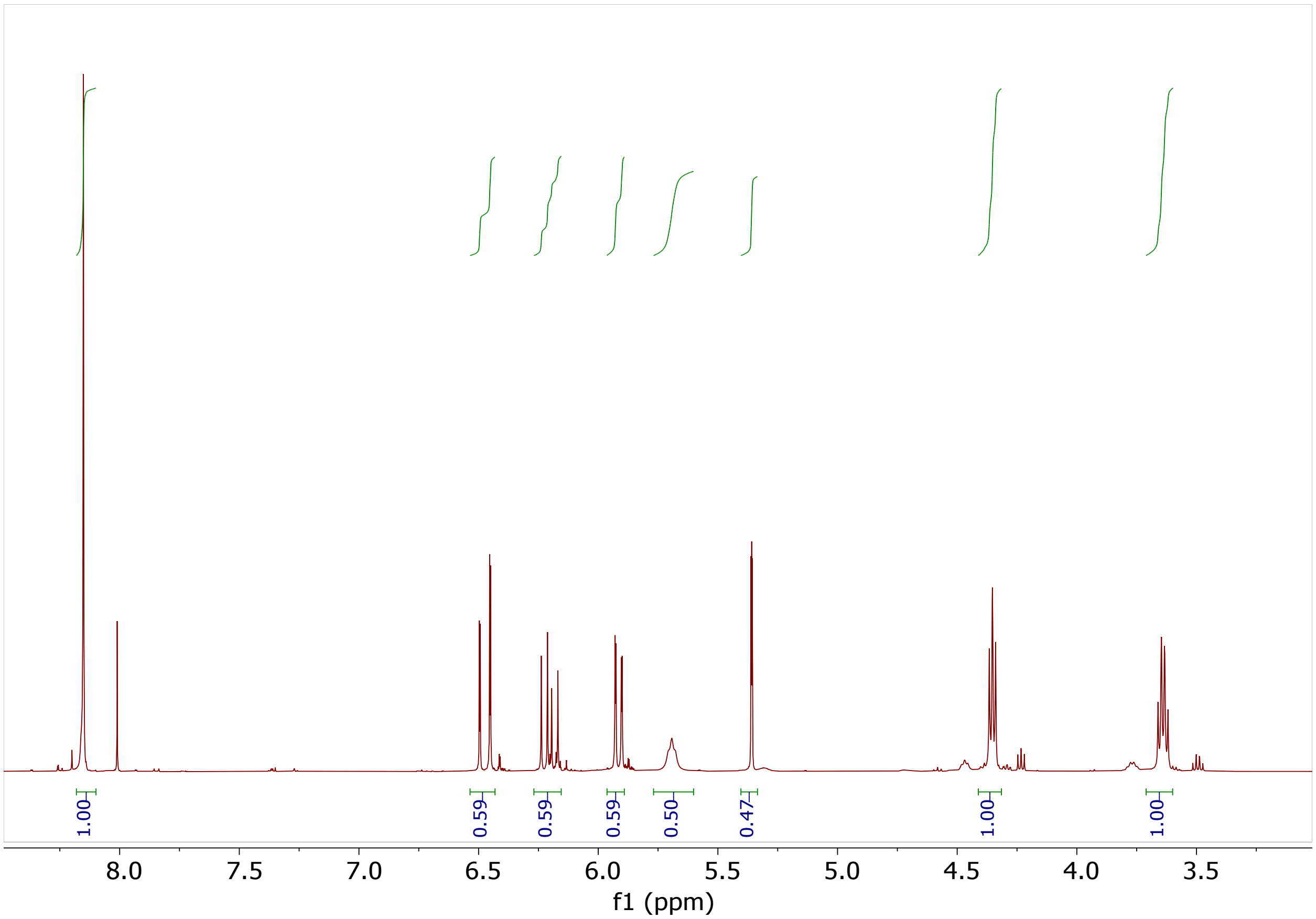

Figure S7: ^{1}H NMR of TIPhUA

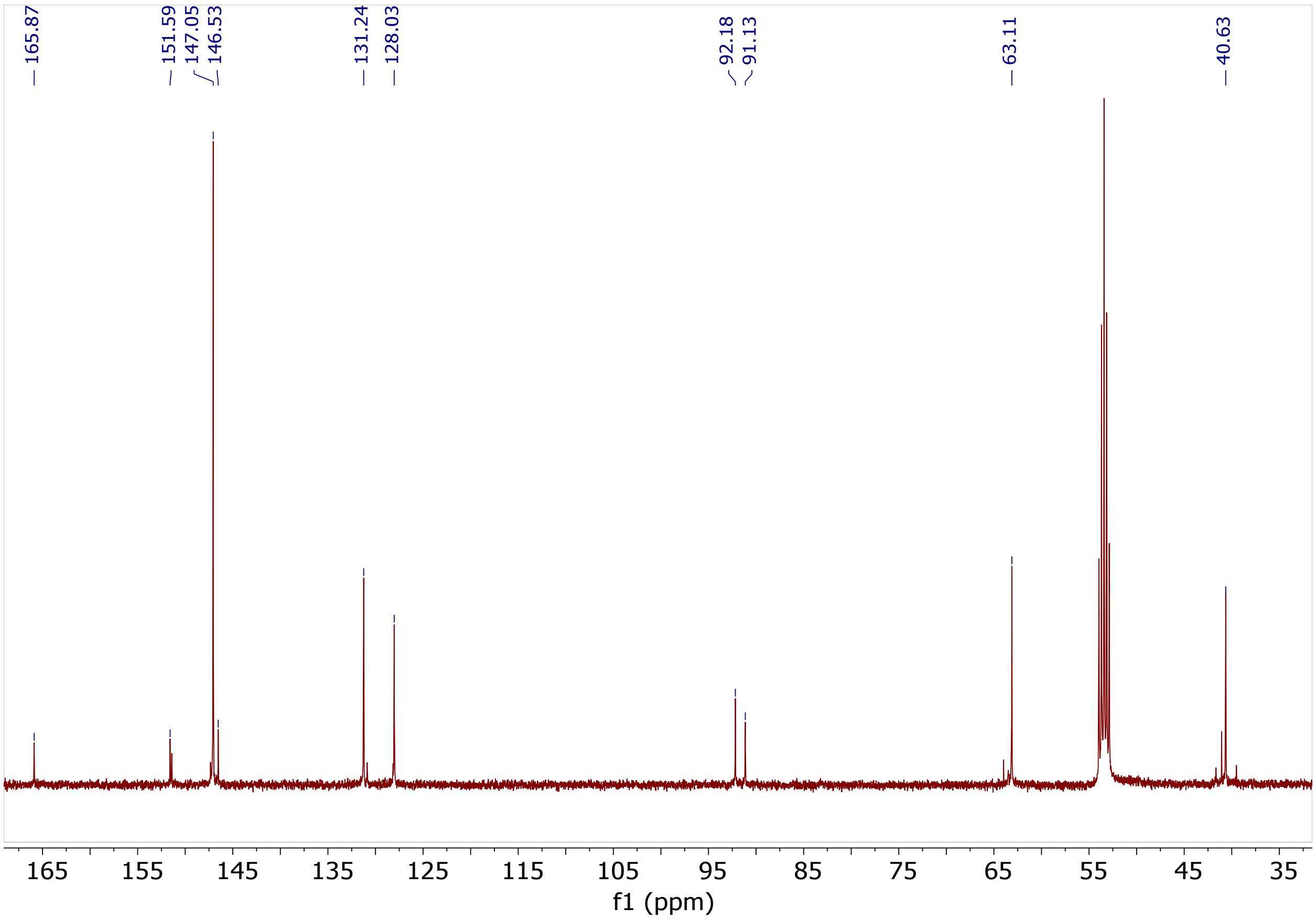

Figure S8: ^{13}C NMR of TIPhUA

3. Mass Spectroscopy

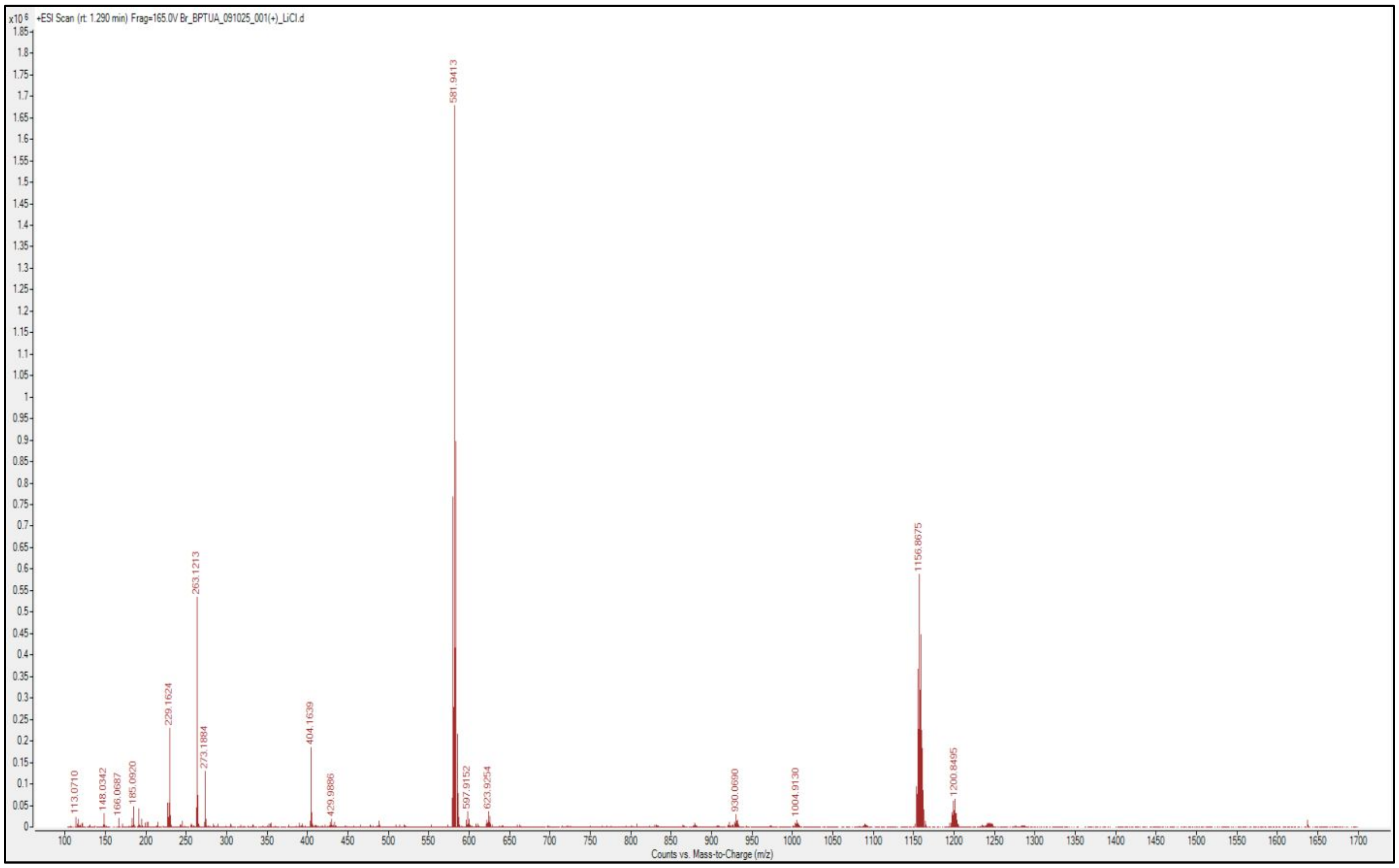

Figure S9: Br-BPTPUA positive polarity Li^+ in 100-1700 m/z range.

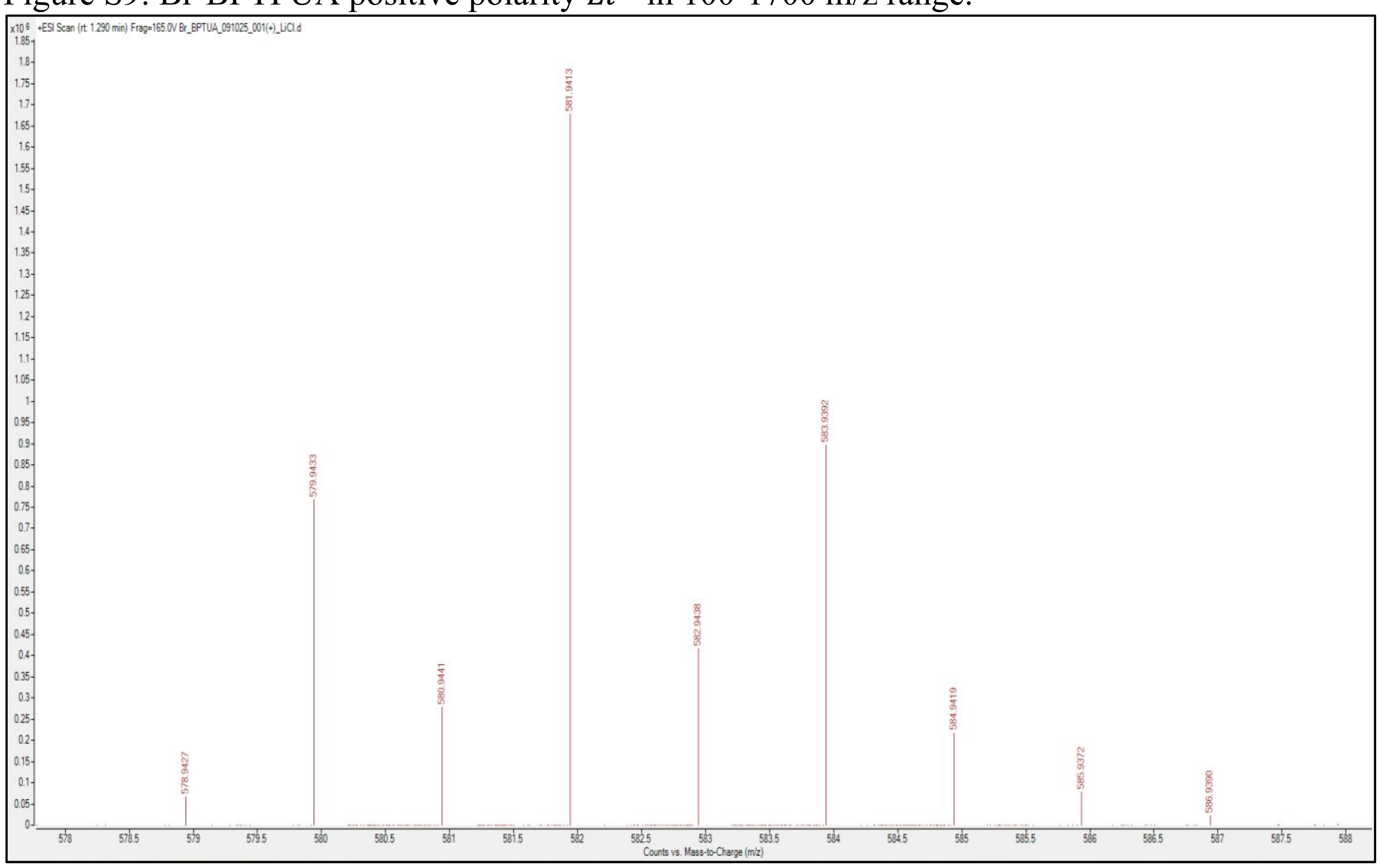

Figure S10: Br-BPTPUA positive polarity Li^+ in 578-588 m/z range. m/z (Li+): 581.94 (100.0%), 582.94 (25.3%), 583.94 (56.6%), 584.94 (11%), 585.94 (2.1%), 586.9 (1.1%).

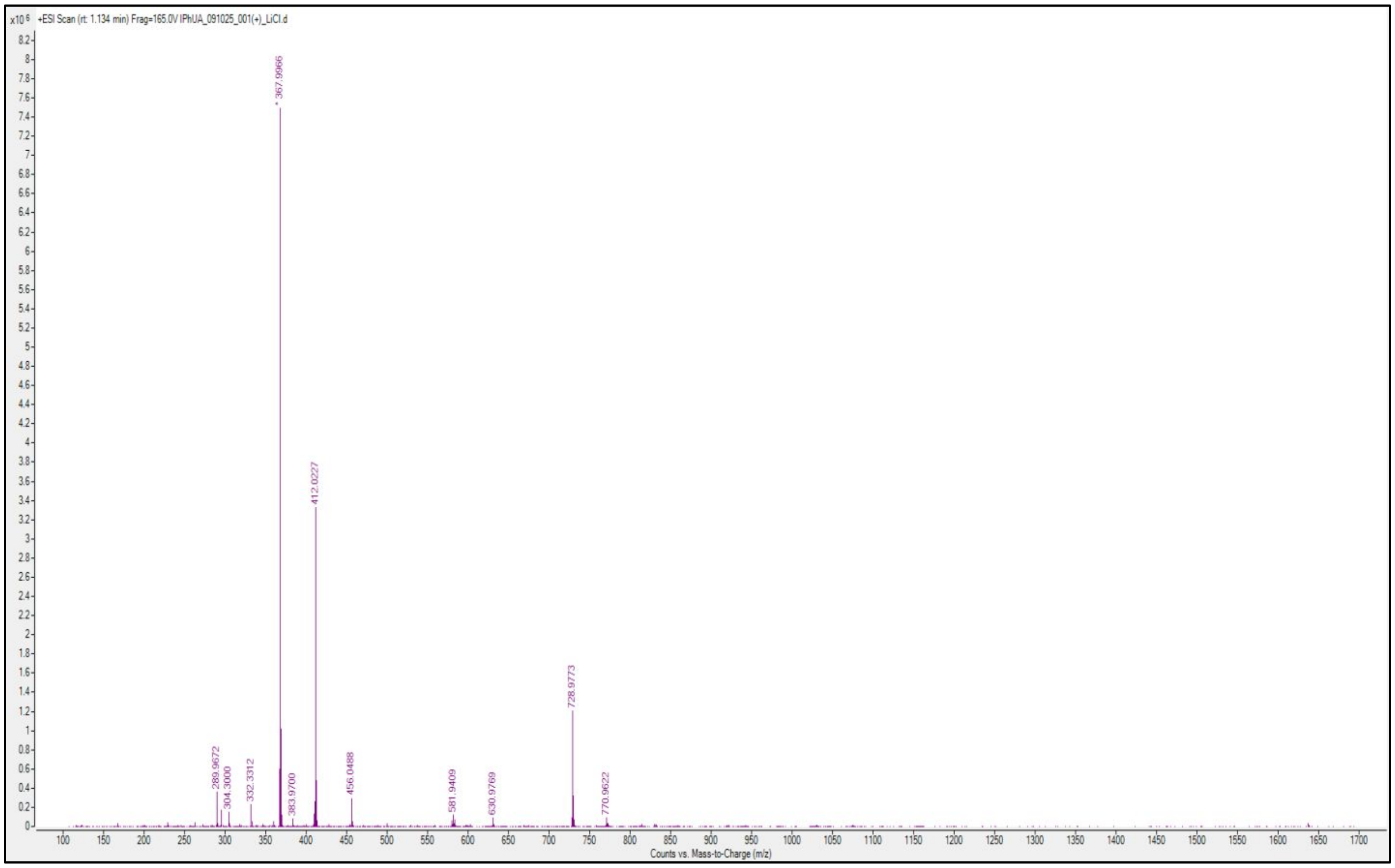

Figure S11: IPhUA positive polarity Li^+ in 100-1700 m/z range.

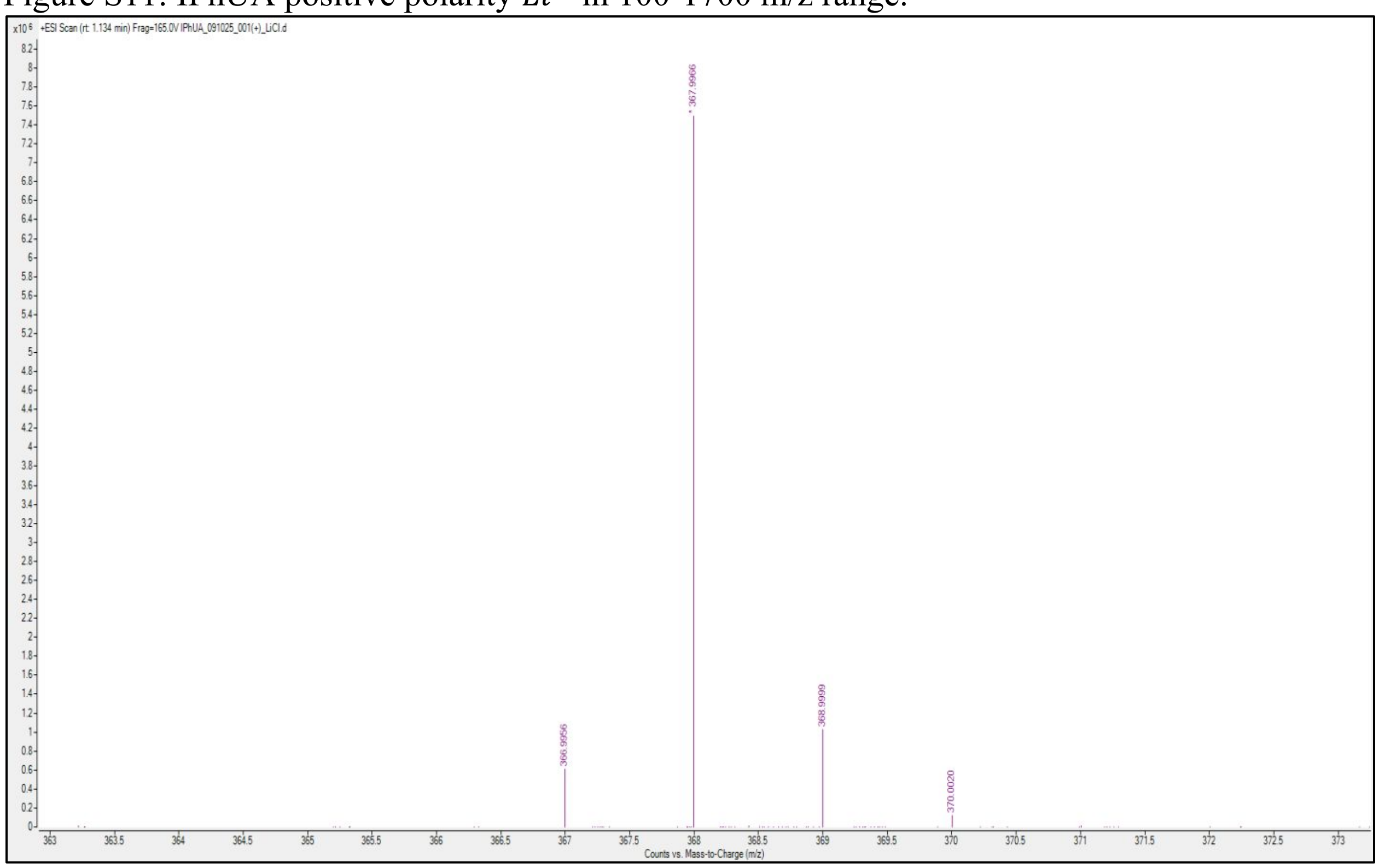

Figure S12: IPhUA positive polarity Li^+ in 363-373 m/z range. m/z (Li+): 367.99 (100.0%), 368.99 (13.3%), 370.00 (1.6%).

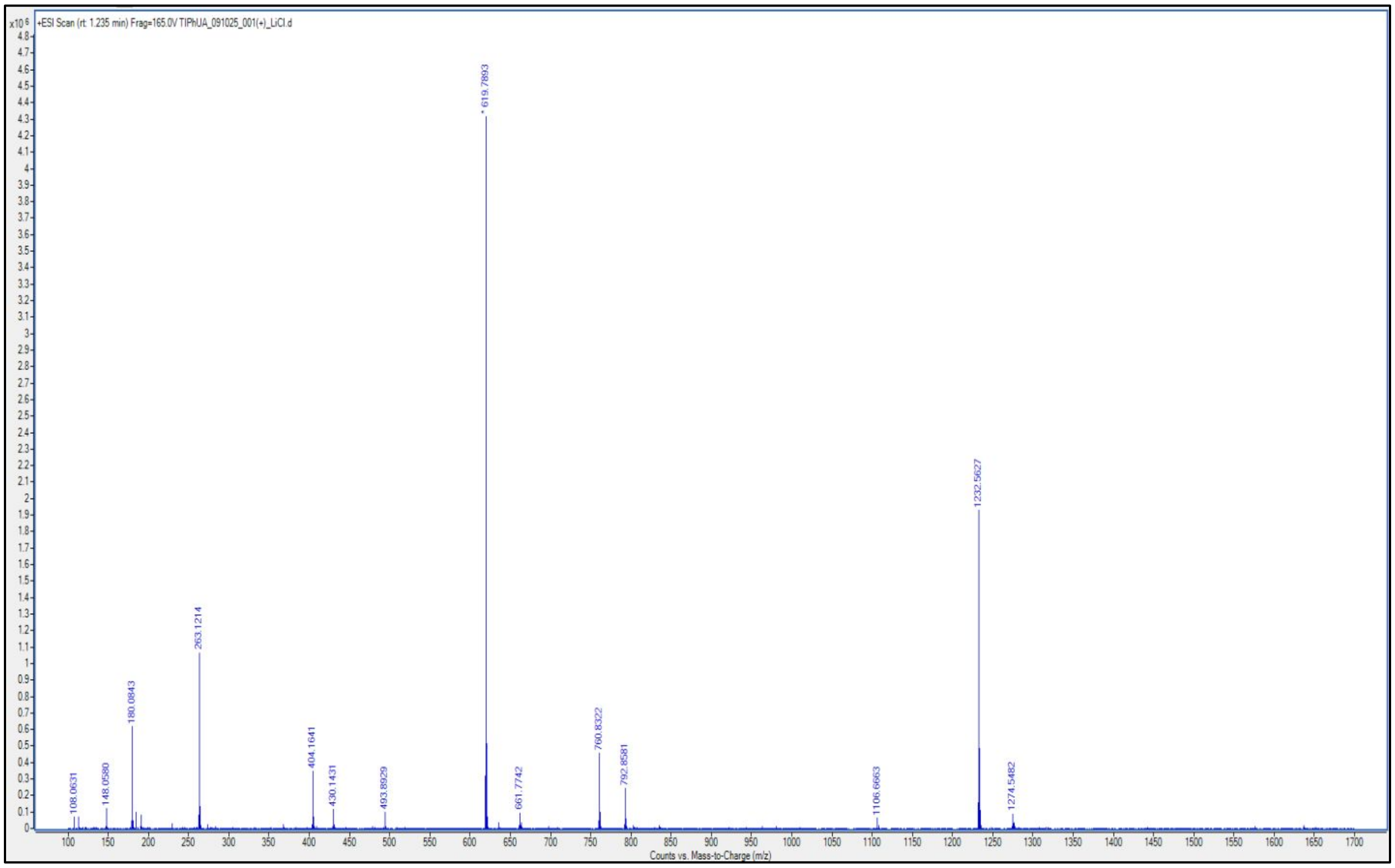

Figure S13: TIPhUA positive polarity Li^+ in 100-1700 m/z range.

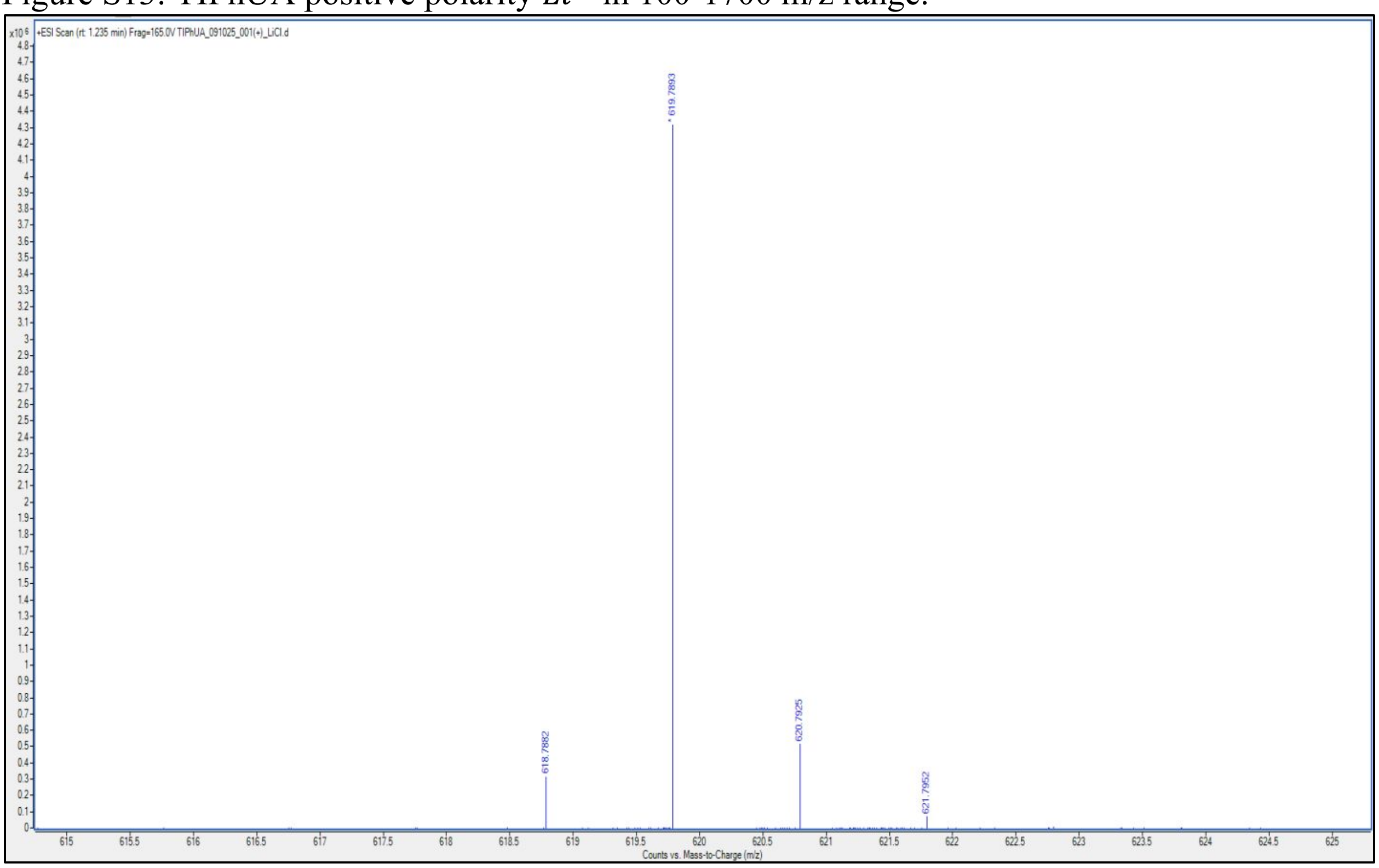

Figure S14: TIPhUA positive polarity Li^{+} in 615-625 m/z range. m/z (Li+): 619.79 (100.0%), 620.79 (13.3%), 621.79 (0.5%).

4. Solubility parameters derived from Hansen Solubility Parameters in Practice software

Table S1: Solubility parameters (δ_d,δ_p,δ_h) of the 1st stage polyurethane and 2nd stage monomers.

	$\delta_d\ (MPa^{0.5})$	$\delta_p\ (MPa^{0.5})$	$\delta_h\ (MPa^{0.5})$
BPTPUA	20.1	6.9	5.4
Br-BPTPUA	20.9	6.5	5.7
IPhUA	21.6	6.2	5.9
Polyurethane	18	7.8	4.6

5. 2-stage refractive index calculations

The number density and polarizability terms in the Lorentz-Lorenz relation can be expanded as the summation of the refractive index n_k weighted by volume fraction ϕ_k for each constituent species k,

$$\frac{4\pi}{3}N\alpha = \frac{(n_m)^2-1}{(n_m)^2+2}\phi_m + \frac{(n_P)^2-1}{(n_P)^2+2}\phi_p + \frac{(n_u)^2-1}{(n_u)^2+2}\phi_u \text{S1)}$$

The volume fraction of each component was calculated from the volume change of the formulation upon fractional volume shrinkage of the HRI monomer σ at full conversion c given the initial volumes occupied by the monomer V_{m0} and polyurethane V_{u0} as

$$\frac{\Delta V}{V} = \frac{c\,\sigma\,V_{m0}}{V_{m0}+V_{u0}} = \frac{c\,\sigma}{1+(1-\phi_{m0})/\phi_{m0}} \text{S2)}$$

where the initial volume fractions of monomer and urethane are related as $\phi_{m0} + \phi_{u0} = 1$. The volume fractions of each component at a particular conversion can then be calculated as

$$\phi_m = \frac{1-c}{1-c\sigma+(1-\phi_{m0})/\phi_{m0}} \text{(S3)}$$

$$\phi_p = \frac{(1-\sigma)c}{1-c\sigma+(1-\phi_{m0})/\phi_{m0}} \text{S4)}$$

$$\phi_u = \frac{(1-\phi_{m0})/\phi_{m0}}{1-c\sigma+(1-\phi_{m0})/\phi_{m0}} \text{(S5)}$$

Note that this calculation assumes that photopolymerization shrinkage is proportional to conversion and not resisted by vitrification, which does not occur due to the rubbery urethane matrix. Only two cases were considered in this work: post cure ($c = 1$, all polymer and urethane)

and pre cure ($c = 0$, all monomer and urethane). This assumes the films reach full conversion when flood cured, which is corroborated by real-time FTIR in section 3.3.

The fractional shrinkage σ of a monomer converting to polymer, assuming polymer modulus does not impede contraction, that is a stress-free environment, was taken to be:

$$\sigma_{SF} = ShrinkageFactor * f * \frac{\rho_m}{M_w} \tag{S6}$$

where the shrinkage factor (22.5 mL/mol for acrylates[1]) represents the change in molar volume upon polymerization, f is the monomer functionality ($f = 1$ for all monomers in this study), ρ_m is the monomer density, and M_w is the molecular weight.

Finally, the initial volume fraction of monomer ϕ_{m0} can be expressed as a function of the initial wt% loading w_m as follows:

$$\phi_{m0} = w_m \frac{\left(\frac{w_m}{\rho_m} + \frac{1 - w_m}{\rho_u}\right)^{-1}}{\rho_m} \tag{S7}$$

where ρ_m and ρ_u refer to the monomer and urethane density, respectively. After substitutions, the refractive index of 2-stage films pre and post cure is expressed as

$$n_{pre\ cure}(w_m) = \sqrt{\frac{-2w_m n_m^2 \rho_u + n_u^2\left((w_m - 1)\left(2 + n_m^2\right)\rho_m - w_m n_m^2 \rho_u\right)}{(w_m - 1)\left(2 + n_m^2\right)\rho_m - w_m\left(2 + n_u^2\right)\rho_u}} \tag{S8}$$

$$n_{post\ cure}(w_m) = \sqrt{\frac{2w_m(\sigma_{SF} - 1)n_p^2 \rho_u + n_u^2\left((w_m - 1)\left(2 + n_p^2\right)\rho_m + w_m(\sigma_{SF} - 1)n_p^2 \rho_u\right)}{(w_m - 1)\left(2 + n_p^2\right)\rho_m + w_m(\sigma_{SF} - 1)\left(2 + n_u^2\right)\rho_u}} \tag{S9}$$

The Lorentz-Lorenz relation can also be used predict the polymer refractive index. To do this, the Lorentz-Lorenz relation was re-written to solve for the neat polymer refractive index $n_{p,\sigma}$ as a function of the neat monomer index n_m and fractional shrinkage σ:

$$n_{p,\sigma} = Re\left(\frac{\sqrt{1 + \frac{8\pi N_p \alpha}{3}}}{\sqrt{1 - \frac{4\pi N_p \alpha}{3}}}\right) \tag{S10}$$

An expression for the number density of polymer N_p as a function of monomer index n_m can be found by solving Lorentz-Lorenz for N_m and multiplying by the fractional shrinkage σ,

$$N_p = N_m \frac{1}{1 - \sigma} = \frac{3\left(n_m^2 - 1\right)}{4\pi\left(2 + n_m^2\right)\alpha} \frac{1}{1 - \sigma} \tag{S11}$$

Substitution of N_p results in the predicted neat polymer index $n_{p,\sigma}$ as a function of the fractional shrinkage σ and neat monomer index n_m,

$$n_{p,\sigma} = \sqrt{\frac{(2+n_m^2)\sigma - 3n_m^2}{(2+n_m^2)\sigma - 3}}. \text{(S12)}$$

6. Refractive index measurements and dn/dT

Table S2: Refractive index dispersion of 15 wt% monomer in polyurethane thin films (250 μm thick) measured at various wavelengths while the sample is held at 30 °C before and after optical flood cure.

	Pre-cure			Post-cure		
Measurement Wavelength (nm)	BPTPUA	Br-BPTPUA	IPhUA	BPTPUA	Br-BPTPUA	IPhUA
405	1.50644	1.50804	1.51093	1.5127	1.51356	1.51743
525	1.49587	1.49733	1.49813	1.49829	1.49943	1.50048
589*	1.4922	1.49355	1.49396	1.49453	1.49549	1.49617
637	1.48999	1.49122	1.4914	1.49241	1.49326	1.49372
984	1.48267	1.48381	1.48405	1.48511	1.48567	1.50705
1546	1.47889	1.47976	1.47987	1.48081	1.48161	1.48178
Abbe # V_d	53.5	52.3	47.7	47	46.7	42.1
Abbe # V_e	53.6	52.5	47.6	46.7	46.5	41.8

* Refractive index at this wavelength was found via interpolating a Cauchy five parameter fit.

Table S3: Refractive index chromatic dispersion of pure BPTPUA monomer, pure BPTPUA polymer, and pure Br-BPTPUA polymer. IPhUA pure polymer was too brittle to characterize.

	Neat monomer	Neat polymer	
Measurement Wavelength (nm)	BPTPUA	BPTPUA	Br-BPTPUA
405	1.61611	1.64408	1.67332
525	1.58747	1.61528	1.64027
589*	1.58043	1.60779	1.63141
637	1.57684	1.60391	1.62688
984	1.56296	1.58972	1.61092
1546	1.55636	1.58361	1.60522

Abbe # V_d	29.2	30.1	27.5
Abbe # V_e	29	29.9	27.3

* Refractive index at this wavelength was found via interpolating a Cauchy five parameter fit.

The refractive index measurements of 15 wt% HRI monomer loading 2-stage films (Table S2) and of selected pure monomer/polymer samples (Table S3) used to create the chromatic dispersion plots (Figure S15A and S15B). BPTPUA was the only monomer that could be characterized in a pure form, as Br-BPTPUA and IPhUA exist as white powders in monomer form. BPTPUA and Br-BPTPUA polymer was formed by dissolving 1 g of the monomer into the solvent dichloromethane (DCM) along with 0.05 g of the thermal initiator azobisisobutyronitrile (AIBN). Excess DCM was added until the solids dissolved. The solution was left uncovered in a 60 °C oven for 24 hours, or until the DCM had evaporated. The same procedure was used to form IPhUA polymer, but the resulting solid was too brittle to characterize.

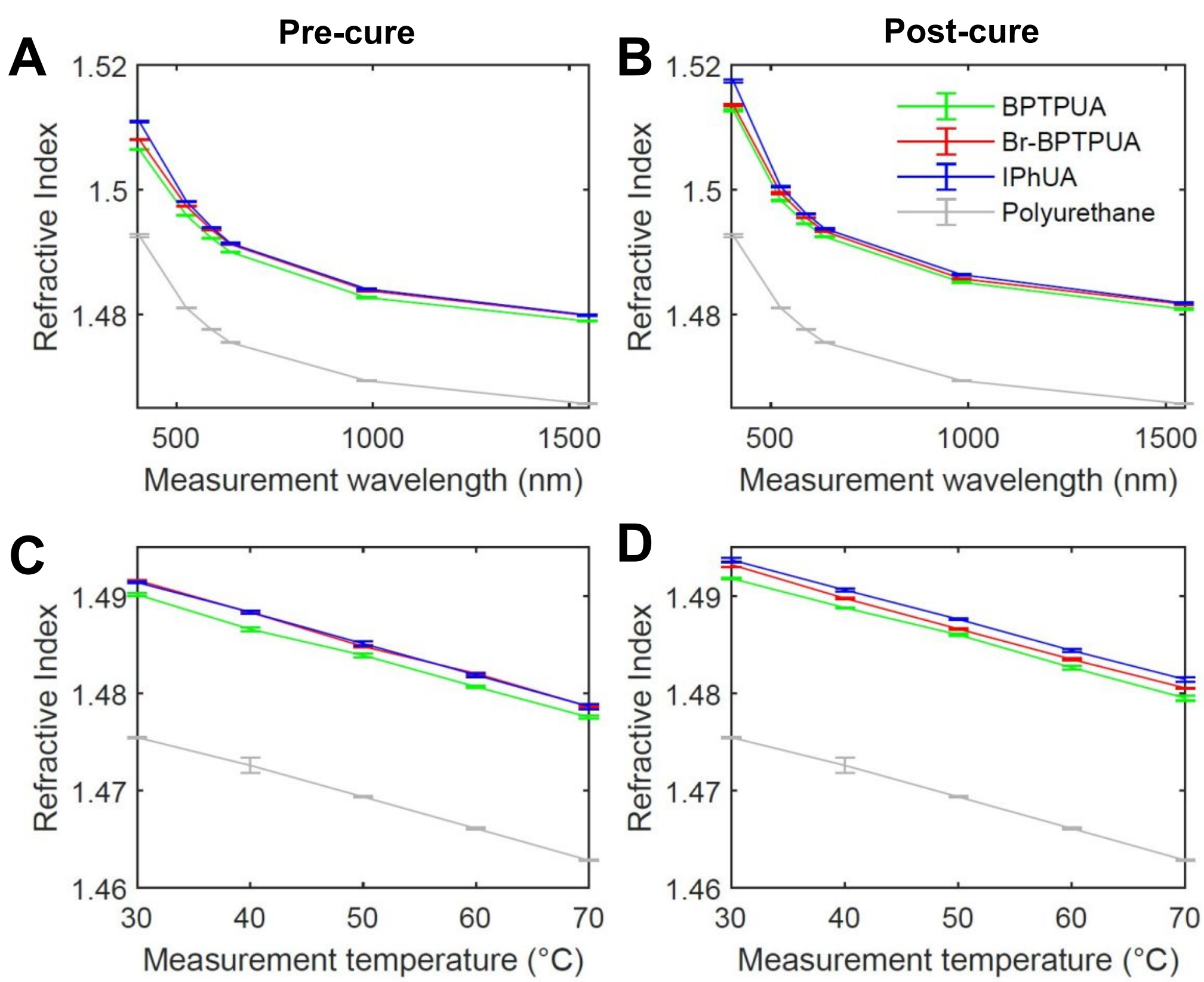

Figure S15: Refractive index measurements of 15 wt% monomer loading in polyurethane films (250 μm) at various wavelengths (T = 30 °C for all dispersion measurements) and at various

temperatures ($\lambda = 637\ nm$ for all dn/dT measurements) pre and post cure. Polyurethane (1st stage) included for completeness. Five measurements were averaged to generate statistics.

The thermo-optic coefficient of refractive index in 2-stage films and polymer measured at 637 nm ranging from 30 °C to 70 °C (Figure S16A) was used to characterize how the refractive index changed with temperature (dn/dT). The IPhUA monomer/polymer and Br-BPTPUA monomer were not characterized, as explained previously. By performing a linear fit to the refractive index as a function of temperature, the thermo-optic coefficient was found. Samples were placed between the coupling head and prism in the Metricon 2010 Prism Coupler and heated on both sides to the desired temperature. Once the target temperature was achieved, the film was allowed to equilibrate for 5 minutes. Because the decrease in index vs temperature remained consistent over the temperature range, implying no quantifiable thermal initiation of the 2nd stage HRI monomers, we concluded that the 2-stage films were thermally stable up to 70 °C. This was validated by thermal cycling of the films, which was used to generate the statistics in Figure S16. Each film underwent three thermal cycles, where the sample was left sandwiched between the coupling head and prism during each temperature change and cycle.

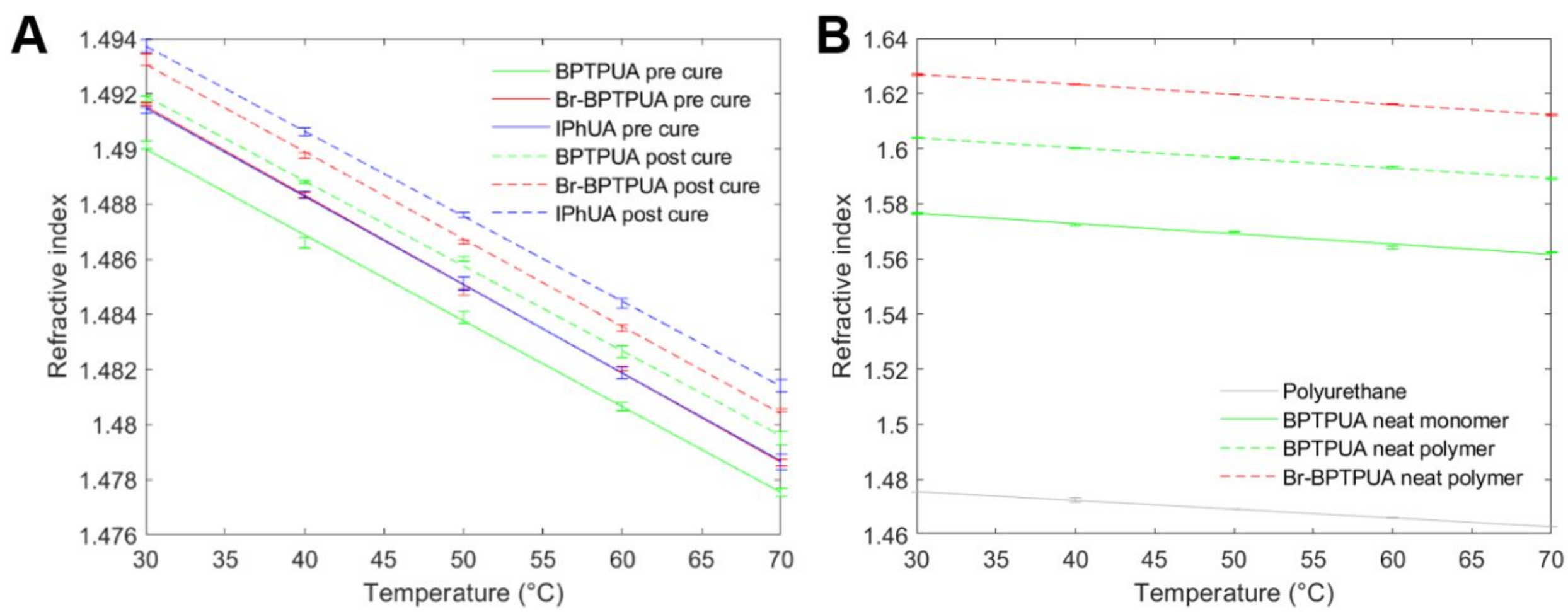

Figure S16: change in refractive index vs temperature of (A) 2-stage 15 wt% HRI loading films and (B) pure BPTPUA monomer/polymer and Br-BPTPUA polymer (1st stage polyurethane included as reference). Linear fits shown with $R^2 \geq 0.99$. Statistics generated from independent measurements at each temperature over three thermal cycles.

Pure BPTPUA monomer and pure BPTPUA/Br-BPTPUA polymers were also characterized for stability (Figure S16B). All species had a negative dn/dT (positive thermal coefficient of expansion) that remained unchanged over the temperature range. Surprisingly, the pure BPTPUA monomer showed a steady dn/dt over the temperature range, indicating no notable

thermally-induced polymerization. This is odd because acrylates are well known to auto-polymerize at elevated temperatures in the absence of solvents. However, the constant change in refractive index over temperature of pure BPTPUA monomer clearly indicates that the monomer is stable up to 70 °C.

7. Thin film approximation and $R_p \propto (I[PI])^{\alpha}$

The rate of polymerization dependence on incident light intensity and determination of dominant termination mechanisms follows the analysis performed by Scott et al[2]. The assumptions they made (thin film approximation, constant photoinitiator concentration) are also applicable here, as shown below. The following is a replication of their analysis applied to our material system and has been included for completeness.

The simplified materials model that was fit to real-time FTIR data assumed that intensity did not change through the film thickness, e.g. the attenuation due to photoinitiator absorbance was minimal. This is a reasonable approximation for the 2-stage films presented, as the change in photoinitiator concentration $-\frac{d[PI]}{dt}$ depends on the quantum yield ϕ of the photoinitiator and the number of photons absorbed in the volume $I_{absorbed}$ as follows:

$$-\frac{d[PI]}{dt} = \frac{\phi I_{absorbed}}{N_a} \text{(S13)}$$

where N_a is Avogadro's number.

Substituting for I using Beer-Lambert's law, $I(z) = I_0 10^{-\epsilon z c}$, where ϵ is the molar absorptivity at the incident irradiation wavelength, z is the film thickness, and c is the concentration, we can simplify to:

$$-\frac{d[PI]}{dt} = \frac{I_o \phi}{N_a}\left(1 - e^{-\ln(10)\,\epsilon z[PI]}\right).\text{(S14)}$$

This expression can then be Taylor expanded about the exponential:

$$-\frac{d[PI]}{dt} = \frac{I_o \phi}{N_a}\left(1 - \left[-\ln(10)\,\epsilon z[PI] + \frac{\left(-\ln(10)\,\epsilon z[PI]\right)^2}{2} + \ldots\right]\right) - \frac{d[PI]}{dt},$$

$$\approx \frac{I_o \phi}{N_a}\ln(10)\,\epsilon z[PI].\text{(S15)}$$

The last statement is true because $1 \gg \ln(10)\epsilon z[PI]$, which is the case here with $z = 250\ \mu m$, $\epsilon_{TPO,\,365nm} = 500\ L\,mol^{-1} cm^{-1}$, and $[PI] = 0.00031\ mol/L$. This is the thin film

approximation. Integration reveals an exponential decay in [PI] with respect to time with a characteristic half-life:

$$[\mathrm{PI}] \approx [\mathrm{PI}]_0 10^{-\frac{\phi\epsilon I_0\lambda}{N_a hc}t} \rightarrow t_{\frac{1}{2}} = \frac{\log(2)\, N_a hc}{\phi\epsilon I_0 \lambda}, \text{(S16)}$$

where h is the Plank constant, c is the speed of light, and λ is the incident light wavelength. Based on the highest intensity used in real-time FTIR ($I_0 = 80\ \mathrm{mW/cm^2}$), the shortest photoinitiator half-life was slightly over 4 minutes, which far exceeded the time required to reach full conversion of monomer to polymer. Therefore, [PI] can be treated as a constant.

Through the steady-state approximation, the rate of polymerization R_p can be related to the photoinitiator concentration [PI] and incident light intensity I to a power α as follows:

$$R_p \propto \left(I_0[\mathrm{PI}]\right)^{\alpha} \text{(S17)}$$

The value of α depends on the dominant termination mechanism, where α→0.5 for bimolecular termination and α→1 for unimolecular termination. To extract this value, the logarithm of both sides can be taken, and a linear fit to $\ln\left(R_p\right)$ vs $\ln\left((I_0[\mathrm{PI}])^{\alpha}\right)$ has a slope of α with constant offset ln ($[\mathrm{PI}]^{\alpha}$).

8. DMA of 15wt% films and of increased loading films

Table S4: Material properties of 2-stage 15 wt% HRI monomer in polyurethane films as determined by DMA. The broadening of the $tan(\delta)$ peak (FWHM increases) upon flood cure indicates increased inhomogeneity, as expected from chain growth polymerization.

	Pre cure		Post cure	
Monomer	T_g (°C)	tan(δ) FWHM (°C)	T_g (°C)	tan(δ) FWHM (°C)
BPPTUA	-49.1 ± 0.6	11.1 ± 1.1	-44.8 ± 1.2	24.2 ± 3.7
Br-BPTPUA	-47.6 ± 0.5	10.9 ± 1.3	-45.2 ± 1.1	24.7 ± 6.2
IPhUA	-47.3 ± 1.4	12.2 ± 0.9	-46.9 ± 1.5	26.3 ± 2.7

While all 2-stage films were mechanically characterized with 15 wt% HRI monomer loading (Figure S17) to compare how each species changed the bulk properties, summarized in Table S4, BPTPUA and Br-BPTPUA were also tested at 30 wt% loading (Figure S18). The higher

wt% loading characterization confirmed that increased monomer presence would not dramatically change the bulk T_g or storage modulus. Unsurprisingly, the breadth of the tanδ peak increased for both monomers pre and post flood cure as compared to films with lower loading, which is expected due to increased inhomogeneity at higher loading. This is especially true post flood cure, where the higher wt% loading films exhibited a dramatic widening of the tanδ peak. As mentioned previously, the broadening of the tanδ peak can be attributed to a combination of expected inhomogeneity due to chain growth polymerization creating a distribution of species with a range of molecular weights, and unwanted PIPS. Based on UV-Vis data and haze measurements, which show extremely small changes in optical properties upon flood cure, it is unlikely (but not impossible) that PIPS plays a major role in broadening the tanδ peak for BPTPUA or Br-BPTPUA.

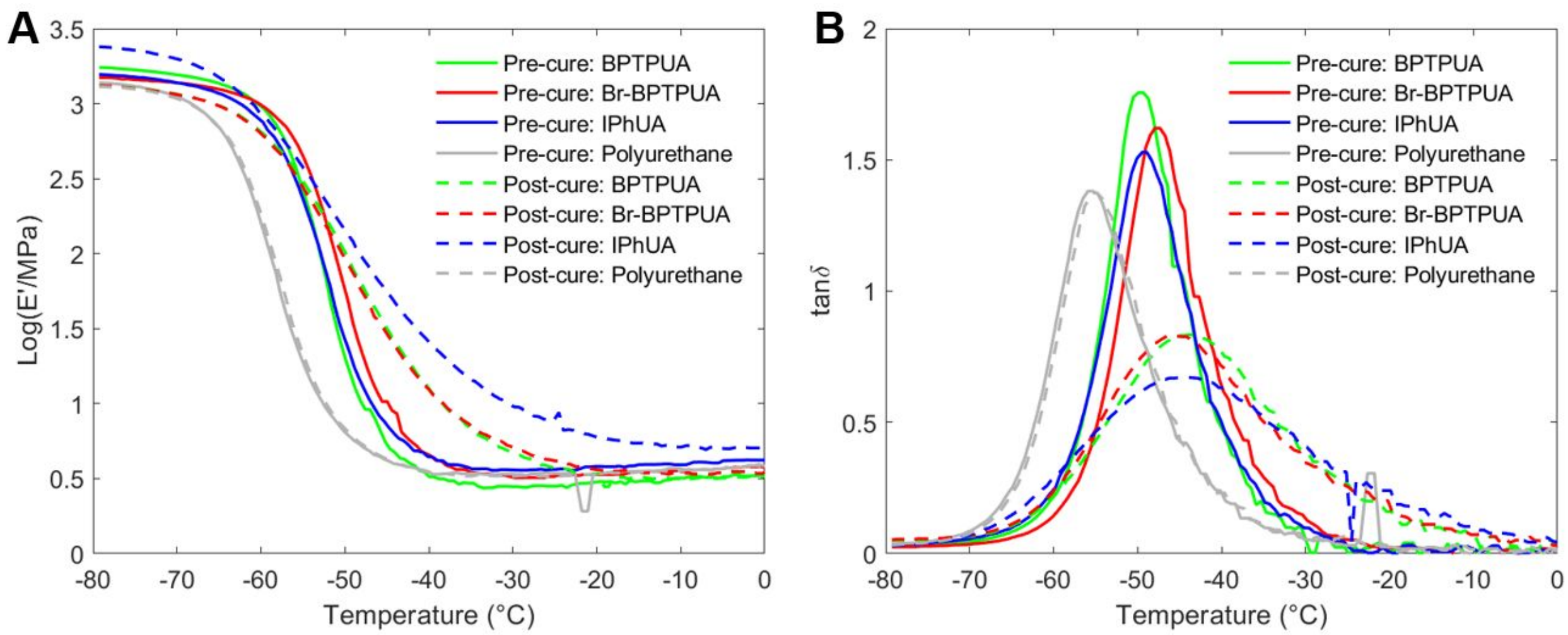

Figure S17: Representative temperature dependence of storage modulus (E') and tanδ of 15 wt% halogenated monomer loading in 2-stage thin films pre and post optical flood cure (1st stage polyurethane included as reference). Polymerization of the monomers during flood cure increases the T_g while widening tanδ, demonstrating increased inhomogeneity. A moving median nearest-neighbor smoothing function was applied to plotted curves.

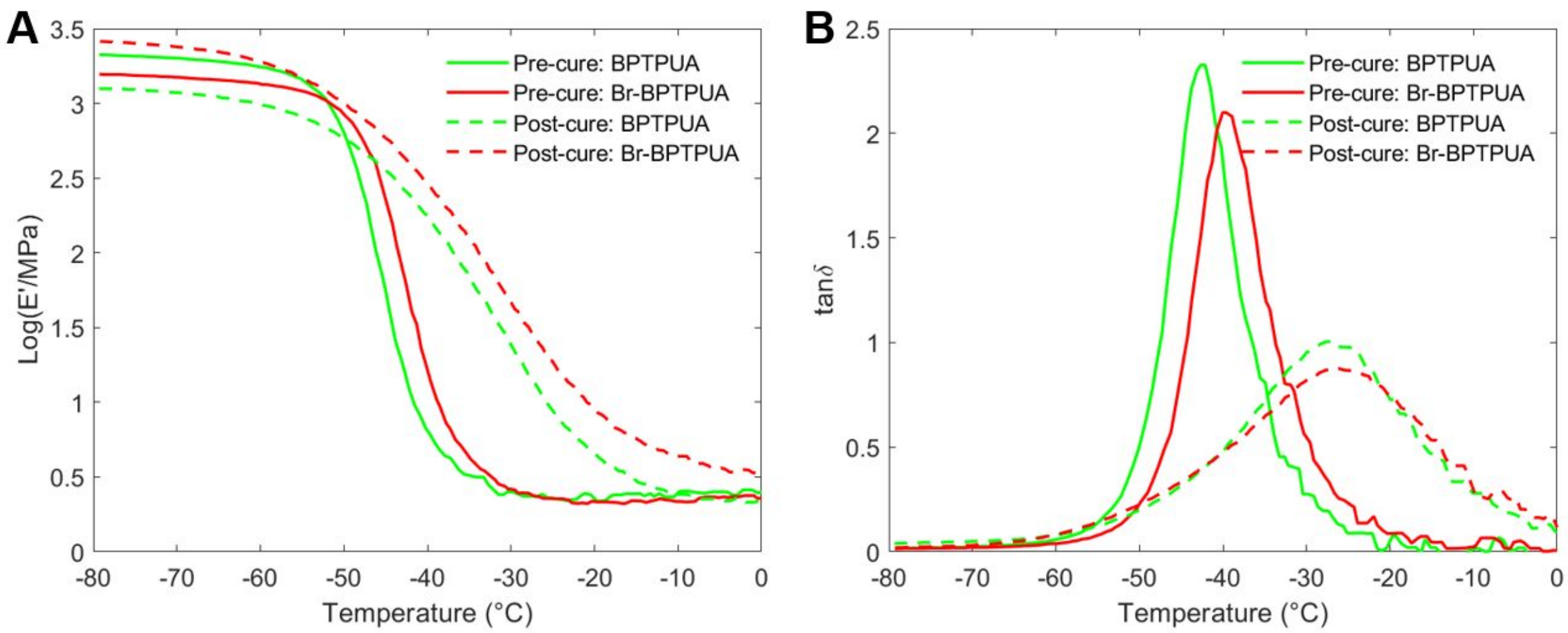

Figure S18: Mechanical properties of 30 wt% 2-stage BPTPUA and Br-BPTPUA 250 μm films pre and post optical flood cure. (A) the log of the storage modulus E' and (B) tanδ.

9. Transparency and haze

Table S5: Transparency and haze pre and post cure of 15 wt% monomer loading 2-stage 250 μm thick films. Polyurethane included for completeness.

	Pre cure		Post cure	
	Transparency (%)	Haze (%)	Transparency (%)	Haze (%)
Polyurethane	100	0 ± 0.1	100	0 ± 0.1
BPTPUA	100	0.1 ± 0.1	99.7 ± 0.1	0.1 ± 0.1
Br-BPTPUA	99.9 ± 0.2	0.2 ± 0.1	99.7 ± 0.2	0.1 ± 0.1
IPhUA	97.7 ± 0.1	5.9 ± 0.1	97.3 ± 0.1	7.7 ± 0.4

10. Resolution test analysis

$$\text{Microscope resolution} = \frac{0.6\lambda}{\text{NA}} \quad \text{(S18)}$$

Table S6: Resolution (smallest negative feature) and critical dimension (smallest positive feature) of 15 wt% HRI monomer 2-stage films photopatterned with the 2-photon patterning system resulting from fits to resolution test patterns.

2-stage material feature	BPTPUA	Br-BPTPUA	IPhUA
Resolution (µm)	0.68 ± 0.13	0.55 ± 0.14	0.64 ± 0.12
Critical dimension (µm)	0.65 ± 0.09	0.58 ± 0.13	0.98 ± 0.1

The results of 2-photon patterning were characterized by brightfield images of the patterned region using a 488 nm light source. This wavelength was chosen to not induce further polymerization, as it is well beyond the absorption band of TPO. The resolution test consisted of two parts: positive feature size and negative feature size. The smallest positive feature was determined patterning blocks of decreasing width, with the smallest block being one pixel in width (pixel width = 0.42 μm). The smallest negative feature size was found by inverting the previous pattern, where increasingly smaller regions were left unexposed while the rest of the film was polymerized, with the smallest unexposed region being one pixel in width.

Analysis of the brightfield images was performed in MATLAB and utilized the positive/negative change in intensity caused by sharp changes in refractive index (Figure S19). Three separate resolution test patterns were generated on each film and the resulting image slices were averaged to mitigate the effects of unwanted particulates in the 2-stage material or on the glass surface. The 2D image was then averaged along the y-direction to generate an intensity modulation plot, which was then fit to using the "findpeaks" function in the signal processing toolbox. After the peak locations were identified, the smallest positive feature was defined as the smallest distance between a positive and negative intensity modulation peak on the positive pattern, and similarly for the smallest negative feature. Critically, the brightfield images were able to discern all patterned structures, indicating the 2-stage films have sub-micron spatial resolution. The calculated resolution of Br-BPTPUA is below the resolution of the microscope, but it should be noted that the upper-bound of the uncertainty in this derived value is still within the measurement range of the instrument.

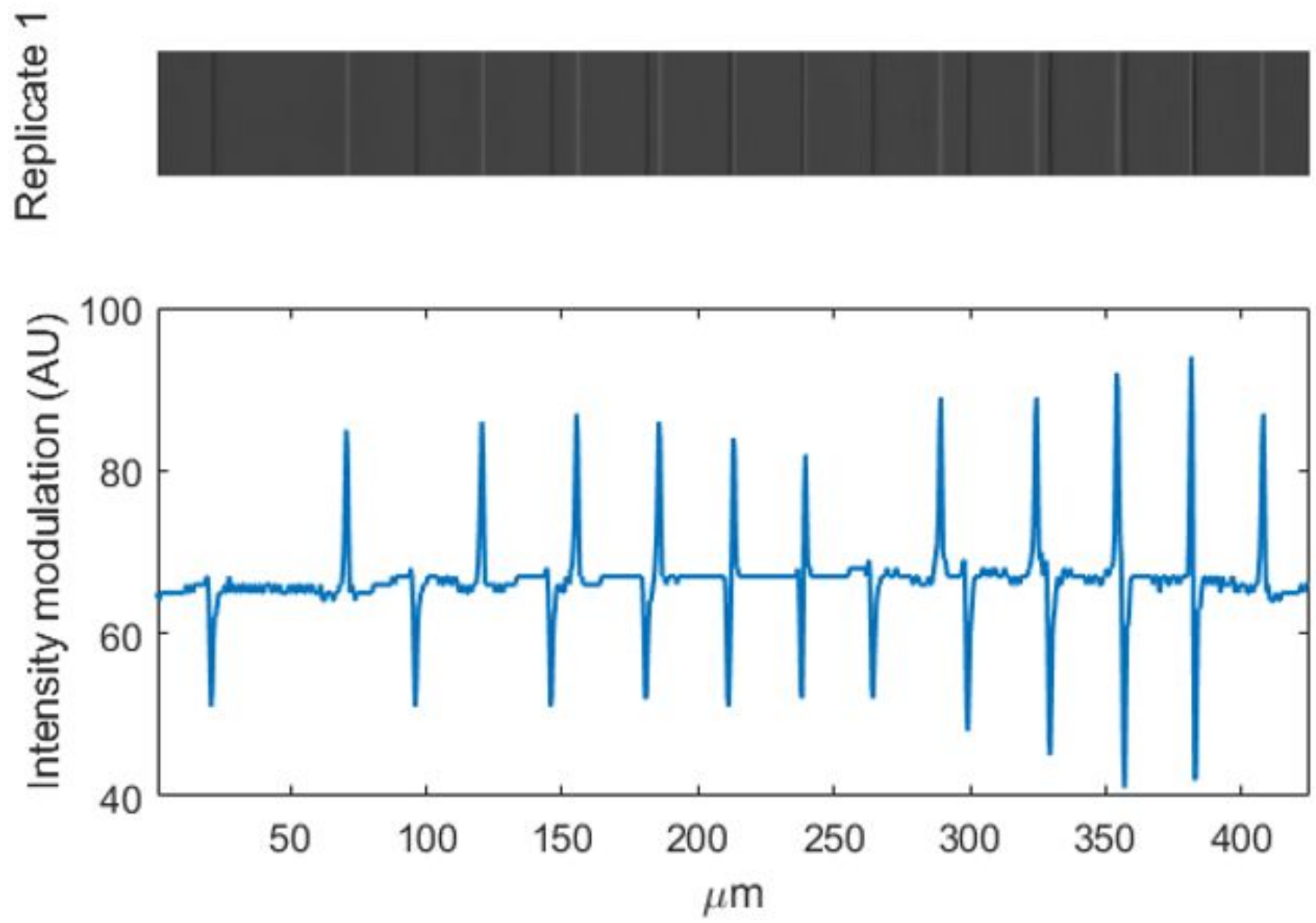

Figure S19: Example of intensity modulation peaks due to sharp changes in refractive index from patterns with sub-micron spatial resolution. Starting from the left, positive features were patterned with decreasing rectangle thickness (smallest positive feature is 0.42 μm), followed by decreasing thickness negative features.

11. Holography recording and readout setup

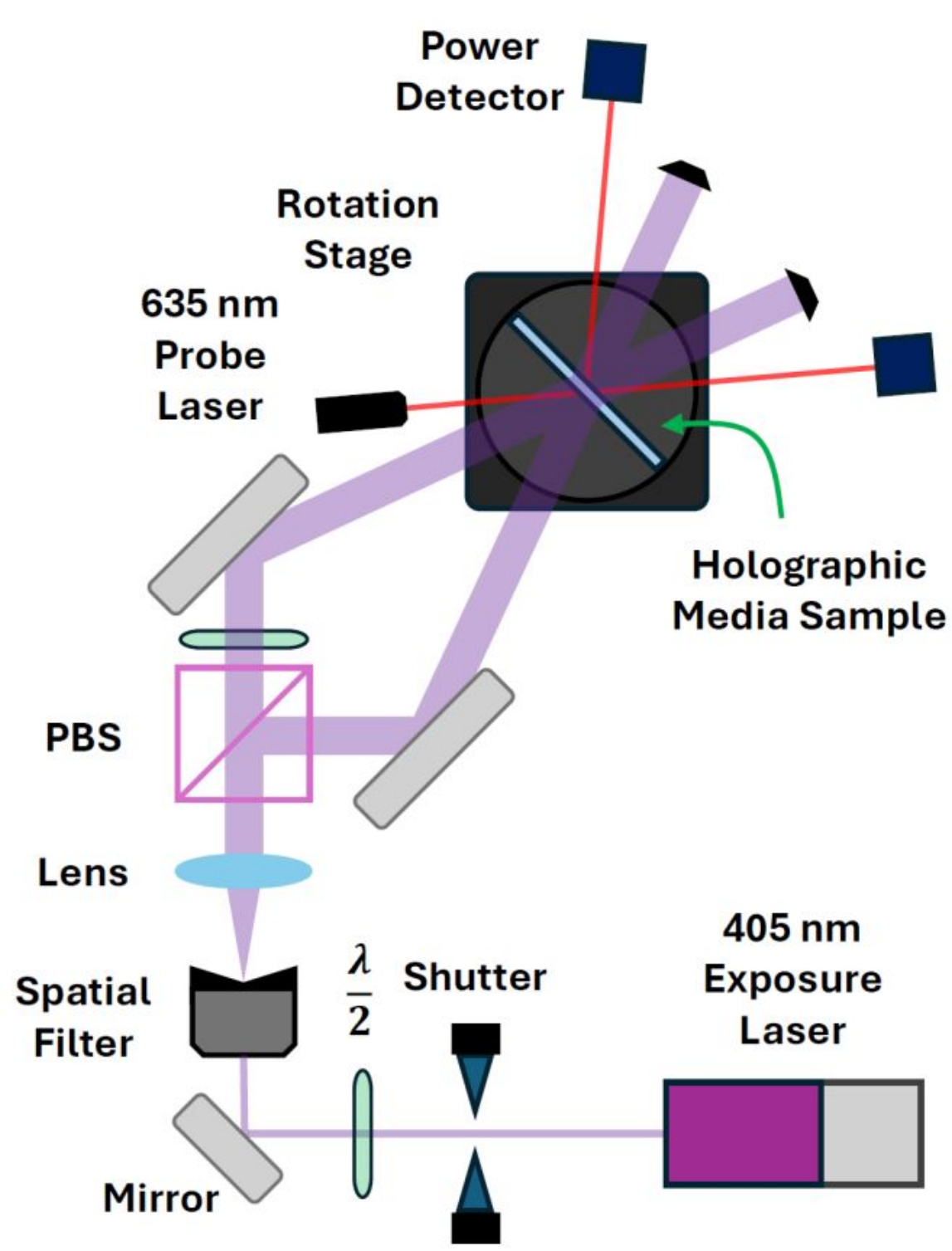

Figure S20: Diagram of holographic media recorder and readout.

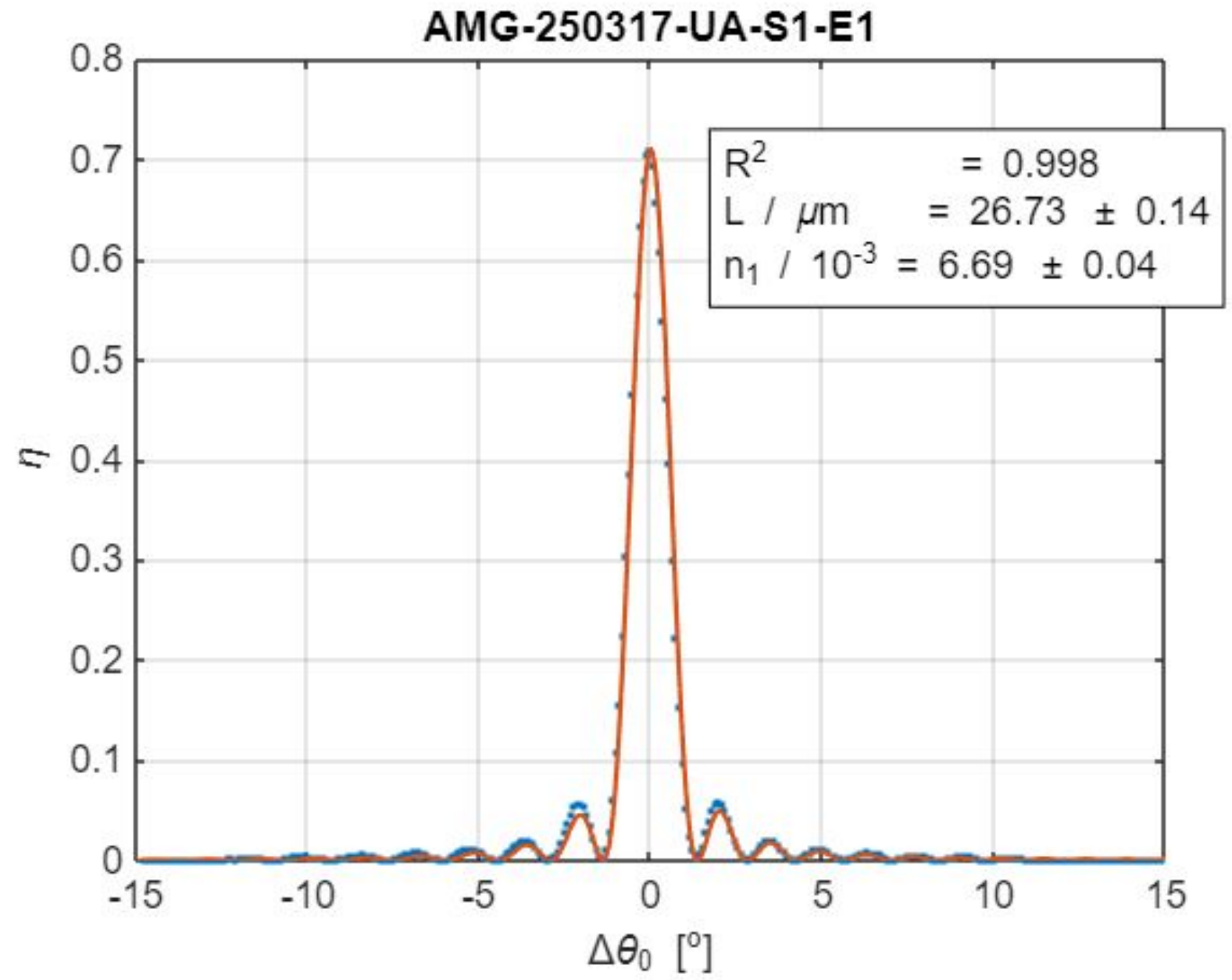

Figure S21: angular selectivity and fit to CWT of a diffraction grating recorded in a 15 wt% BPTPUA 2-stage film.

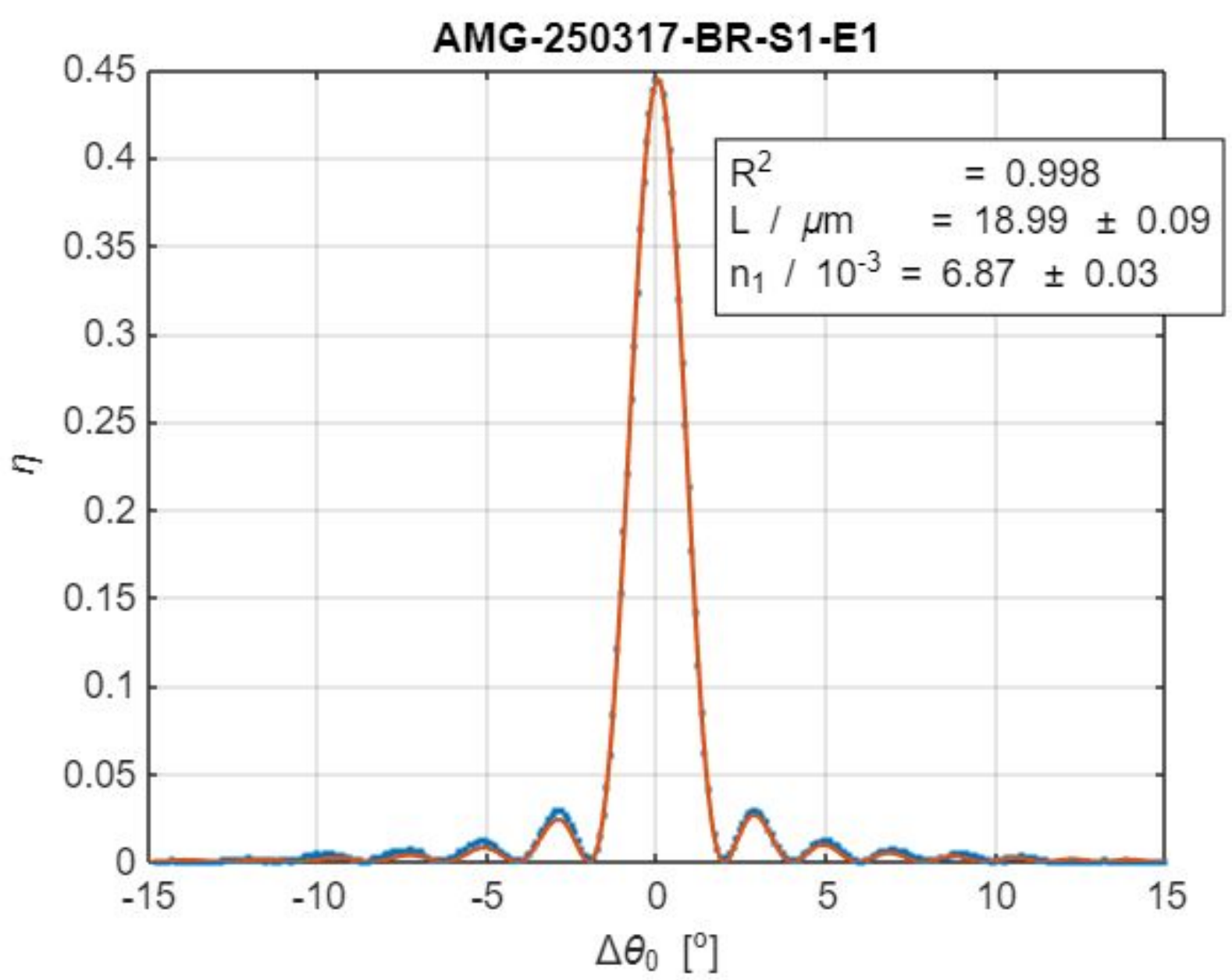

Figure S22: angular selectivity and fit to CWT of a diffraction grating recorded in a 15 wt% Br-BPTPUA 2-stage film.

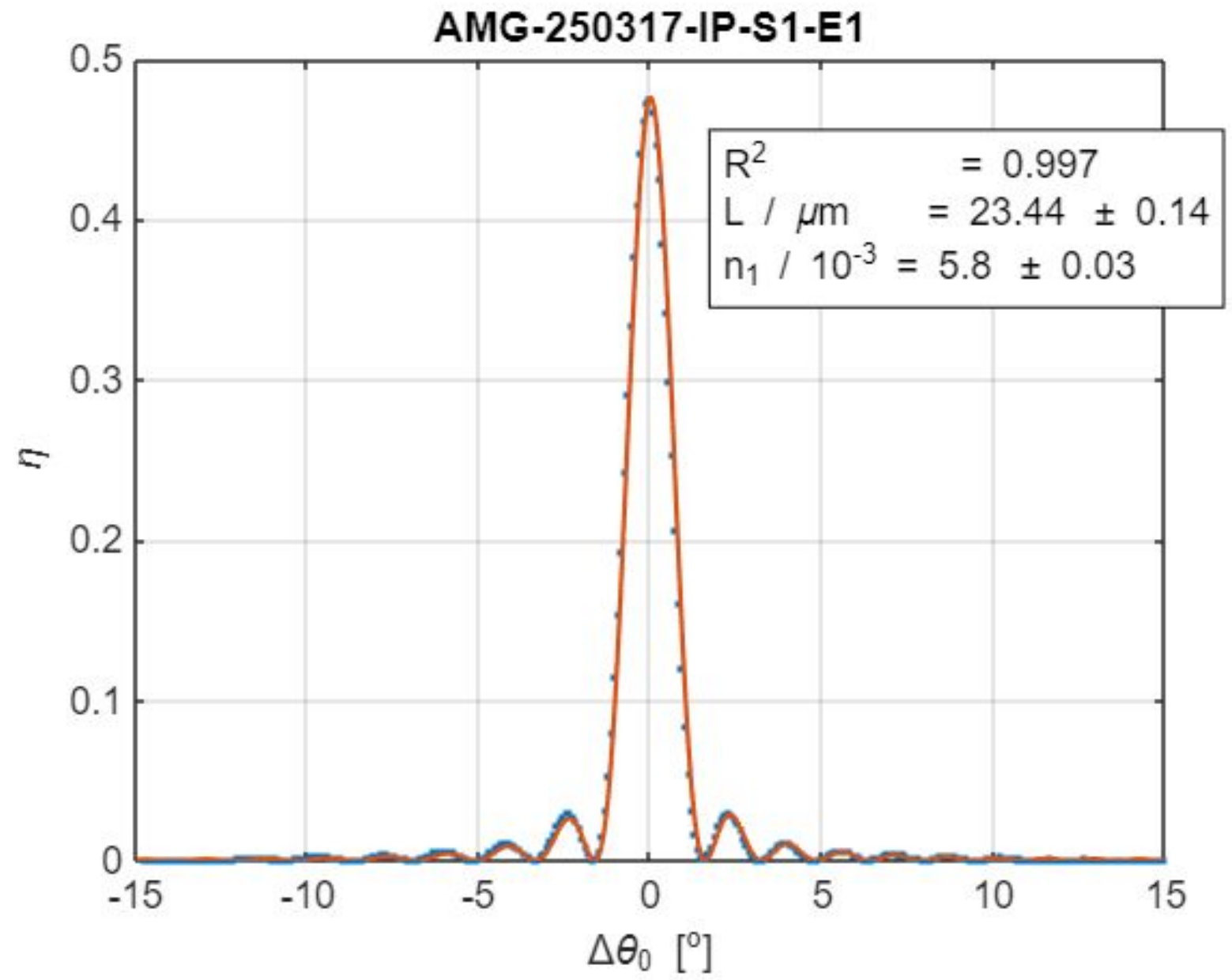

Figure S23: angular selectivity and fit to CWT of a diffraction grating recorded in a 15 wt% IPhUA 2-stage film.

References:

(1) Patel, M. P.; Braden, M.; Davy, K. W. M. Polymerization Shrinkage of Methacrylate Esters. *Biomaterials* **1987**, *8* (1), 53–56. https://doi.org/10.1016/0142-9612(87)90030-5.
(2) Scott, T. F.; Kloxin, C. J.; Draughon, R. B.; Bowman, C. N. Nonclassical Dependence of Polymerization Rate on Initiation Rate Observed in Thiol−Ene Photopolymerizations. *Macromolecules* **2008**, *41* (9), 2987–2989. https://doi.org/10.1021/ma8002505.
